# Probing the fluorination effect on the self-assembly characteristics, *in vivo* fate and antitumor efficacy of paclitaxel prodrug nanoassemblies

**DOI:** 10.7150/thno.61337

**Published:** 2021-07-06

**Authors:** Xin Wang, Bin Yang, Lingxiao Li, Tian Liu, Shiyi Zuo, Dongxu Chi, Zhonggui He, Bingjun Sun, Jin Sun

**Affiliations:** 1 Department of Pharmaceutics, Wuya College of Innovation, Shenyang Pharmaceutical University, Shenyang 110016, China.; 2 School of Pharmacy, China Medical University, 77 Puhe Road, Shenyang 110122, China.; 3 Department of Pharmacy, the First Hospital of China Medical University, 155 Nanjing North Street, Shenyang 110001, China.; 4 School of Pharmacy, Shenyang Pharmaceutical University, Shenyang, 110016, China.

**Keywords:** prodrug self-assembly, fluorination effect, paclitaxel, redox responsive, tumor accumulation

## Abstract

**Rationale:** Small-molecule prodrug nanoassembly is emerging as an efficient platform for chemotherapy. The self-assembly stability plays a vital role on the drug delivery efficiency of prodrug nanoassembly. It is reported that fluoroalkylation could improve the self-assembly stability of amphiphilic polymers by utilizing the unique fluorination effect. But the application of fluoroalkylation on small-molecule prodrug nanoassembly has never been reported.

**Methods:** Here, fluoro-modified prodrug was developed by conjugating paclitaxel with perfluorooctanol (F_8_-SS-PTX), and the paclitaxel-octanol prodrug (C_8_-SS-PTX) was used as control. The fluoro-mediated self-assembly mechanisms were illustrated using molecular dynamics simulation. In addition, the impacts of fluoroalkylation on the pharmacy characters, *in vivo* fate and antitumor effect of small-molecule prodrug nanoassembly were investigated in details.

**Results:** Fluoroalkylation significantly improved the self-assembly stability of F_8_-SS-PTX NPs both *in vitro* and *in vivo*, which could be attributed to the fluoro-mediated hydrophobic force and halogen bonds. The AUC_0-24h_ and tumor accumulation of F_8_-SS-PTX NPs was 6-fold and 2-fold higher than that of C_8_-SS-PTX NPs, respectively. As a result, F_8_-SS-PTX NPs exhibited much better antitumor effect than C_8_-SS-PTX NPs and Abraxane.

**Conclusion:** Fluoroalkylation could improve the self-assembly stability, *in vivo* fate, and antitumor efficacy of small-molecule prodrug nanoassemblies, which could be an effective strategy for the rational design of advanced nanomedicines.

## Introduction

Cancer is a malignant disease with high mortality 1. Chemotherapy remains one of the most effective strategies in the treatment of cancer 2. However, the therapeutic efficiency of chemotherapy is usually restricted by its poor tumor-targeting and severe systemic toxicity 3, 4. For example, paclitaxel (PTX), a typical antimitotic agent, is widely applied for various types of cancer. Unfortunately, the *in vivo* nonselective distribution of PTX caused serious emesis and myelosuppression 5. In addition, due to the extremely low solubility of PTX, Taxol (the clinical used injections of PTX) has to use Cremophor EL and ethanol as solubilizers. The related allergic reaction severely limits the clinical application of Taxol 5.

During the past decades, various strategies had been developed to deal with the current dilemma 6-9. Among them, small-molecule prodrug nanoassemblies, integrating the superiorities of prodrug strategies and nanomedicine, have emerging as advanced platforms for the delivery of chemotherapeutics 10-15. Compared with the traditional nanoparticulate drug delivery systems (nano-DDSs) like liposomes and micelles, the small-molecule prodrug nanoassemblies display extra-high drug loading capacity (usually over 50%) since the prodrugs act as the dual roles of cargoes and carriers. Additionally, prodrug nanoassemblies preclude the application of bio-incompatible materials, then avoiding the toxicity caused by excipients 16, 17. To acquire self-assembling prodrugs, the common strategy is to conjugate PTX with a carbon chain, commonly fatty acid or fatty alcohol 9, 18. For example, Wang et al. developed photoactivatable prodrug cocktail nanoaseemblies for chemo-photodynamic therapy 12. Both cabazitaxel and photosensitizer were conjugated with polyunsaturated fatty acid, and the developed prodrugs could co-assemble into uniform nanoparticles. The introduction of carbon chains makes the prodrug molecules more flexible, which prevents the formation of large aggregates during self-assembly process.

Fluoroalkylation could enhance the self-assembly stability of amphiphilic polymers by utilizing the unique fluorination effect 19-22. Due to the extremely low polarizability of fluorine atom, fluoroalkyls could hardly interact with any other molecules and therefore tend to aggregate into a both hydrophobic and lipophobic phase 23. The strong fluoro-mediated hydrophobic interactions and halogen bonds then drive the self-assembly of fluoroalkyls into stable nanoparticles in water 24, 25. Although fluoroalkylation is recently used to facilitate the self-assembly of amphiphilic polymers 22, 25, the application of fluoroalkylation in the field of prodrug nanoassemblies has not been reported yet. We wonder whether fluoroalkylation could impact the self-assembly stability of small-molecule prodrug nanoassemblies, thereby impacting their *in vivo* fate and antitumor effect.

**Figure 1:**
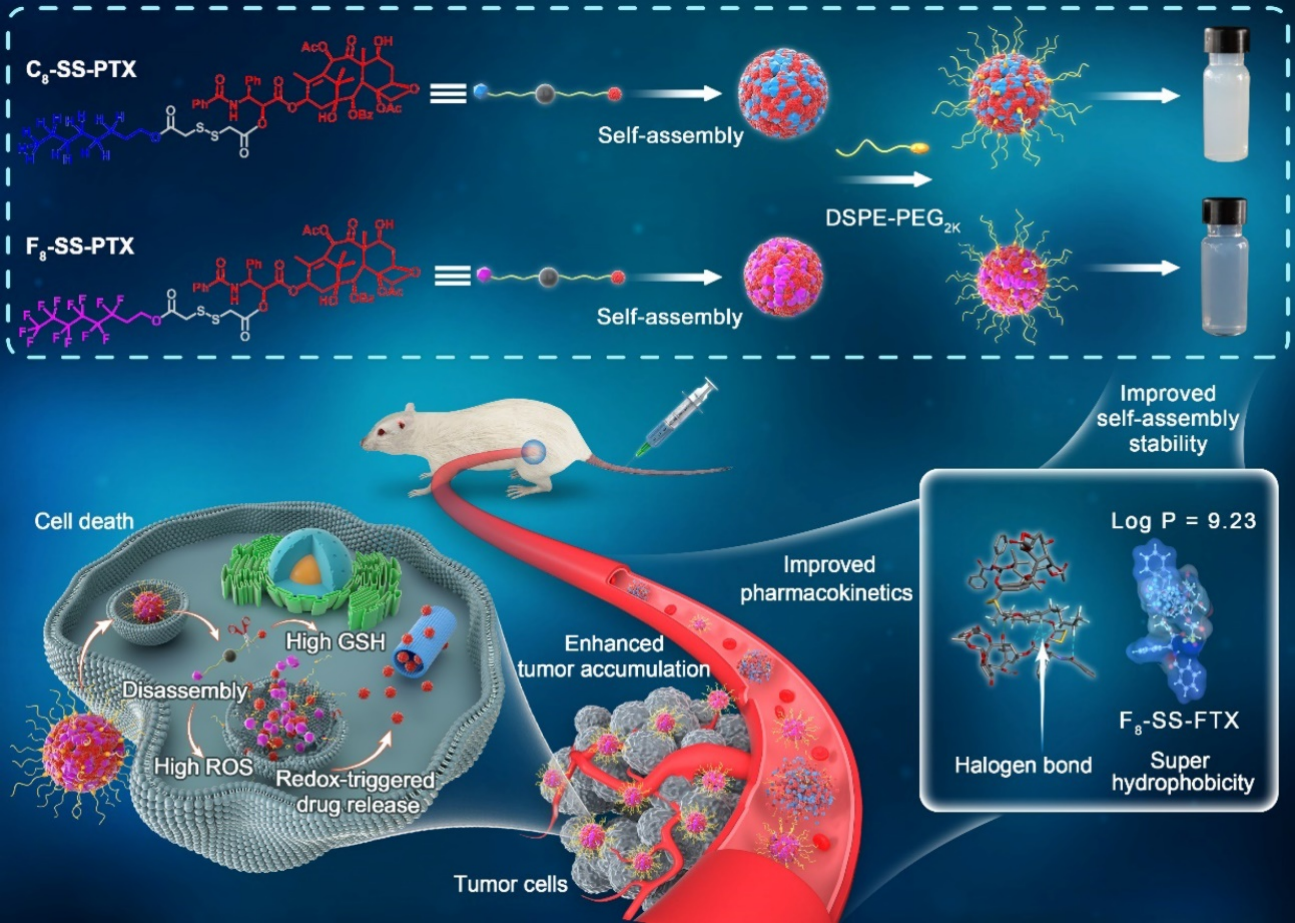
** Schematic illustration of disulfide bond-bridged perfluorooctanol/octanol-PTX prodrug nanoassemblies for cancer therapy.** Compared with C_8_-SS-PTX NPs, the F_8_-SS-PTX NPs greatly improves the self-assembly stability, thereby improving the circulation time and tumor accumulation of prodrug nanoassemblies.

Based on the above hypothesis, herein, fluoro-modified prodrug was developed by conjugating PTX with perfluorooctanol via a disulfide bond, abbreviated as F_8_-SS-PTX. In addition, PTX linked with octanol (C_8_-SS-PTX) was synthesized as a control. Disulfide bond, sensitive to the redox microenvironment in tumor cells, is a common linker to construct stimuli-responsive DDSs 16, 26-29. Both the two prodrugs could form into uniform nanoassemblies, with the drug loading over 50%. For the first time, we found that the fluoroalkylation of carbon chains facilitated the self-assembling capability of PTX prodrugs, thereby improving the stability, pharmacokinetics, tumor accumulation and antitumor activity of small-molecule prodrug nanoassemblies (**Figure 1**).

## Methods

### Materials

PTX, dithiothreitol (DTT), 1,1'-dioctadecyl-3,3,3',3'-tetramethylindotricarbocyanine iodide (DiR), and 2-(4-Amidinophenyl)-6-indolecarbamidine dihydrochloride (DAPI) were supplied from Meilun Biotech (Dalian, China). Perfluorooctanol, octanol, coumarin-6, 1-Ethyl-3-(3-dimethyllaminopropyl) carbodiimide hydrochloride (EDCI), 4-dimethylaminopyrideine (DMAP) and hydrogen peroxide (H_2_O_2_) were all bought from Aladdin Biochemical Technology Co. Ltd. (Shanghai, China). 1,2-distearoyl-sn-glycero-3-phosphoethanolamine-N-methyl (polyethylene glycol)-2000 (DSPE-PEG_2k_) was provided from Shanghai Advanced Vehicle Technology Co. Ltd. (Shanghai, China). Cell culture reagents and 3-(4,5-dimethylthiazol-2-yl)-2,5-diphenyltetrazolium bromide (MTT) were derived from Gibco (Beijing, China). Calcein-AM/PI double stain kit, Annexin V-FITC/PI apoptosis assay kit and Microtubule Tracker Red were purchased from Beijing Solarbio Science & Technology Co., Ltd. TUNEL apoptosis detection kit and Ki67 cell proliferation kit were supplied from Service biotech Co., Ltd. (China). Hoechst 33342 was provided by BD Biosciences (USA). The other reagents used in this paper were of analytical.

### Synthesis of PTX-Perfluorooctanol and PTX-Octanol Prodrugs

The preparation of the two prodrugs was according to the literature 16. In brief, dissolving dithiodiglycolic acid (4 mmol) with 6 mL of acetic anhydride in a flask. Remaining it stirs for 2 h with the protection of nitrogen atmosphere at 25 °C, then, methylbenzene (30 mL) was put into the reaction solution and evaporated under vacuum for several times. After that, 20 mL of anhydrous dichloromethane was immediately poured into the system to dissolve the crude product, and then DMAP (0.4 mmol) was dripped into the solution under stirring. Subsequently, perfluorooctanol or octanol (2 mmol) were added into the system. Remaining the solution stirs for 24 h at 25 °C, then, the silica gel column chromatography (cyclohexane/acetone = 20:1, 1% glacial acetic acid) was used to separate out the product (50% yield). After that, the product (1 mmol) obtained above was dissolved in a flask with dichloromethane and the solution of EDCI (2 mmol) and DMAP (1 mmol) was dripped into the system. Remaining the solution stirs for 2 h in ice bath under the protection of nitrogen, then, PTX (1 mmol) was added into the system and remained the system stirring for 24 h at 25 °C. Then, the crude product was purified using preparative liquid chromatography by utilizing acetonitrile/water (95:5) as the mobile phase (50% yield). NMR spectral analyses (400 MHz for ^1^H) and mass spectrometry were used to certify the prodrug structure. The purity of the prodrugs was measured by high performance liquid chromatography (HPLC).

### Preparation, Characterization and Molecular Dynamics Simulation of Prodrug Nanoassemblies

In brief, using 0.2 mL of ethanol to dissolve 2 mg of prodrugs and 0.5 mg of DSPE-PEG_2k_, and then injecting the mixture solution into 2 mL of purified water with a stirring speed of 1000 r/min. After 5 min, ethanol was removed under vacuum. The non-PEGylated PTX prodrug nanoassemblies were manufactured according to the same method but without the help of DSPE-PEG_2k_. The DiR or coumarin-6-marked nanoassemblies were prepared by making PTX prodrugs with DiR or coumarin-6 co-assemble. In brief, the ethanol solution of prodrugs, DSPE-PEG_2k_, together with DiR or coumarin-6 were injected into purified water with stirring. The Zeta potential and particle size of PTX prodrug nanoassemblies were determined by Zetasizer (Nano ZS, Malvern Co., UK). Transmission electron microscopy (TEM, JEOL, Japan) was utilized to certify the morphologies of the PTX prodrug nanoassemblies.

The drug loading (%) = MW_PTX_/MW_prodrug_ × (M_prodrug_/(M_prodrug_+M_DSPE-PEG2K_)) × EE%. MW represented the molecular weight, M represented weight and EE% represented encapsulation efficiency. The EE% was obtained by the following methods: in brief, 1 mL of prodrug nanoassemblies (1 mg/mL) was put into an ultrafiltration centrifuge tube (MW = 3000 Da), and centrifuged at 4000 rpm for 20 min. Then 1 mL of water was added to redisperse the nanoparticles. 20 μL of the redispersed nanoparticles was withdrawn and mixed with 180 μL of acetonitrile to determine the peak area of prodrugs by HPLC. The peak area was regarded as A1. Meanwhile, mixing 20 μL of the prodrug nanoassemblies with 180 μL of acetonitrile to determine the peak area of prodrugs by HPLC. The peak area was regarded as A2. The encapsulation efficiency was obtained by A1/A2 × 100%.

The molecular dynamics simulation was utilized to elucidate the self-assembly mechanism. In brief, using Gaussian view 5 to construct the two monomer prodrug molecules. Then, the structures were initially optimized based on the semi-empirical AM1 method, and the process was executed based on the Gaussian09 program. Thereafter, the Packmol program was used to construct 2 initial model systems composed of 20 molecules. The GAFF force field was used to generate the parameters. Using the TIP3P water box model, the side length of the water box was set to 1 nm. First, perform 5000 steps of energy minimization, and then perform short-time 100ps simulation under NVT and NPT ensemble respectively, and finally generated 100ns balance simulation. The cutoff radius was adjusted to 1.0 nm, the time step was adjusted to 2 fs, and the temperature and pressure are set to 300 K and 1 bar, respectively. Using the autodock program to do molecular docking of two C_8_-SS-PTX and two F_8_-SS-PTX systems respectively, and obtain the binding energy. Utilizing Discovery Studio program to analyze hydrogen bonding and hydrophobic interaction. All molecular dynamics simulations were calculated based on the Gromacs2018 program.

### Colloidal Stability

To study the impact of fluoroalkylation on the stability of prodrug nanoassemblies, the non-PEGylated and PEGylated formulations were placed at room temperature for 7 days. At the planned time points, the mean diameters were determined using a Malvern Zetasizer. Further, the PEGylated prodrug nanoassembly solution was incubated in PBS solution (pH 7.4) supplement with 10% fetal bovine serum (FBS) for 48 h at 37 °C with gentle shaking. At pre-determined time points, the mean diameter was determined. Moreover, the storage stability of the PEGylated formulations at 4 °C for 14 d was also tested. To investigate the change in particle size of prodrug nanoassemblies in culture medium, we put 100 μL of nanoassemblies into 900 μL of culture medium (pH 7.4 and pH 5.0). At pre-determined time points, the mean diameter was measured using Zetasizer.

### Redox-Responsive Release

The *in vitro* release profiles were studied using PBS solution (pH 7.4) supplemented with 30% ethanol as the releasing medium, which could meet the sink condition, namely possessing enough capacity to dissolve the hydrophobic PTX released from F_8_-SS-PTX or C_8_-SS-PTX 16, 17. In brief, 0.2 mL of prodrug nanoassemblies (200 nmol) were poured into 30 mL of the releasing medium containing different concentrations of dithiothreitol (DTT, a prevailing GSH simulant) or hydrogen peroxide (H_2_O_2_, a prevailing ROS simulant) and shocked at 37 °C. 0.2 mL of the releasing medium was withdrawn at the designated time. Then, the content of PTX or prodrug (F_8_-SS-PTX or C_8_-SS-PTX) was measured using HPLC (n = 3 for each group). To clarify the result of drug release, the formulation was incubated with release medium containing DTT or H_2_O_2_ for 30 min with gentle shaking, and the generated intermediates were certified using UPLC-MS/MS (Waters Co., Ltd., Milford, MA, USA) and LCMS-8060 (Shimadzu Co., Japan), respectively. In addition, the changes of these nanoassemblies in the presence of DTT and H_2_O_2_ were monitored by TEM.

### Cell Culture

Mouse colon cancer cells (CT26), mouse mammary carcinoma cells (4T1), non-small-cell carcinoma cells (A549) and human liver cells (L02) were supplied by the cell bank of Chinese Academy of Sciences (Beijing, China). CT26 cells, 4T1 cells and L02 cells were grown in medium composed of Gibco 1640, 10% FBS and 1% antibiotics. A549 cells were grown in DMEM containing 10% FBS and 1% antibiotics. All cells were placed in incubator at 37 °C under a 5% CO_2_ atmosphere.

### Cell Uptake

CT26 cells were seeded in glass dishes and cultured for 24 h. Then, the culture medium was discarded, and fresh medium containing free coumarin-6 or coumarin-6-marked formulations at a dose of 250 ng/mL equal to coumarin-6 was added, and further cultured for 0.5 h or 2 h. Then, using cold PBS to wash cells three times, aiming to clear away the coumarin-6 that has not entered the cell, and fixed with 4% formaldehyde. Subsequently, the cells were washed again, and stained with Hoechst 33342. Finally, the samples were watched using confocal laser scanning microscopy (CLSM, TCS SP2/AOBS, LEICA, Germany). For quantitative analysis, CT26 cells were seeded in 12-well plates at a density of 1 × 10^5^ cells per well for 24 h. The cells were washed, collected and re-suspended in PBS after incubation with free coumarin-6 or coumarin-6-labeled prodrug nanoassemblies for 0.5, 2, 8, 24 h. Untreated cells were utilized as control. Cellular uptake was analyzed by flow cytometry on a FACS Calibur instrument (Becton Dickinson).

### Cytotoxicity Assays

Cell viability assays were evaluated using the MTT assay on CT26 cells, 4T1 cells, A549 cells and L02 cells. Briefly, the 96-well plates were used to culture cells (1000 cells/well) for 24 h. Then, the medium was discarded, and various concentrations of Taxol or prodrug formulations diluted by fresh medium was added to incubate for 48 h. Cells treated with blank medium were regarded as the negative control (n = 3 for each group). Then, 40 μL of MTT (5 mg/L) solution was placed into the plates and stayed for 4 h at 37 °C. After that, the medium in plates was replaced with DMSO (180 μL). The absorbance at 490 nm was recorded using a microplate reader (Thermo Scientific, USA). Using Graph Pad Prism 8 to calculate the IC_50_ values. The tumor selective index (SI) value was gained by dividing the IC_50_ values of L02 cell lines by the IC_50_ values of tumor cell lines 30, 31.

### AM/PT staining and apoptosis assay

With regards to AM/PI staining of the living/dead cells, CT26 cells were plated in 12-well plates (5 × 10^4^ cells/well) for 24 h, Then, the medium was discarded, and Taxol or prodrug nanoassemblies diluted by fresh medium was added to incubate for 48 h (200 nmol/L equivalent to PTX). After that, cells were treated according to the procedure suggested in the Calcein-AM/PI apoptosis detection kit. And then, the cells were observed under an inverted fluorescence microscope to distinguish between the dead and living cells. For apoptosis assay, CT26 cells were plated in 12-well plates at a density of 1 × 10^5^ cells/well and incubated for 24 h. Then, the medium was replaced with fresh RPMI-1640 medium containing Taxol or prodrug nanoassemblies with a PTX equivalent of 200 nmol/L. After that, the cells were washed three times with PBS. Then, the cells were collected and treated according to the procedure suggested in the Annexin V-FITC/PI apoptosis detection kit. Apoptosis was measured by flow cytometer (Becton Dickinson, USA) and the results were analyzed using FlowJo 7.6 software.

### *In vitro* microtubule polymerization assay

CT26 cells were grown in glass dishes and cultured for 24 h. Then, the culture medium was discarded, and the fresh medium containing Taxol or prodrug nanoassemblies (100 nmol/L equivalent to PTX) was added, and further cultured for 48 h. Then, the cells were washed by PBS for three times, and fixed with 4% formaldehyde. After that, the cells were treated according to the procedure suggested in Microtubule Tracker Red detection kit. Subsequently, the cells were washed again, and stained with Hoechst 33342. Finally, the samples were observed using confocal laser scanning microscopy (CLSM, TCS SP2/AOBS, LEICA, Germany). Images were analyzed using Image J software.

### Intracellular Drug Release

CT26 cells were grown in 24-well plates (2 × 10^5^ cells/well) and cultured for 24 h. Then, the cells were exposed to Taxol or prodrug formulations at doses of 50, 100 or 200 nmol/L equal to PTX for 48 h. Subsequently, collecting the cells and the culture medium to analyze the content of free PTX after sonication and centrifugation via UPLC-MS-MS (ACQUITY UPLCTM, Waters Co., Ltd., Milford, MA, USA).

### Animal Studies

All animal experiments were performed according to the Guide for Care and Use of Laboratory Animals of Shenyang Pharmaceutical University.

### Hemolysis assay

5 mL of blood was taken from the orbit of SD rats and centrifuged for 10 min at 1200 rpm. After that, the supernatant was removed, and then the blood cells in the lower layer were washed with saline until the supernatant was colorless, and obtained a 2% blood cell suspension. Then, mixing saline, prodrug nanoassemblies or Triton X-100 with the above blood cell suspension to make the concentration of the prodrug nanoassemblies or Triton X-100 as 0.5 mg/mL and placing them at 37 °C for 2 h. The samples were centrifuged for 10 min at 3000 rpm and observe whether the supernatant was hemolysis.

### *In vivo* Pharmacokinetic Study

The *in vivo* pharmacokinetic behavior of prodrug formulations was carried out on male Sprague-Dawley rats. The animals were intravenously administered Taxol or PTX prodrug nanoassemblies (5 mg/kg equivalent to PTX, n = 5 for each group). At the planned time intervals, the plasma was acquired by centrifuging the blood samples isolated from orbital to determine the content of prodrugs and free PTX. The chemical stability of prodrug nanoassemblies in the fresh rat plasma was investigated. Briefly, mixing 50 μL of prodrug nanoassemblies with 450 μL of fresh rat plasma, and then putting the mixture in a constant temperature shaker at 37 °C with a shaking speed of 100 r/min. At predetermined intervals, 50 µL samples were withdrawn and 150 µL acetonitrile was added. After vortex and centrifugation, the concentration of the prodrugs in the supernatants was determined by HPLC.

### *In vivo* Biodistribution

BALB/c mice bearing CT26 tumor were used to study the biodistribution of prodrug nanoassemblies via fluorescence imaging. Briefly, DiR solution or DiR-marked PTX prodrug nanoassemblies were intravenously administrated (1 mg/kg equal to DiR), when the tumor volume was about 400 mm^3^. At 4, 12 and 24 h after injection, the mice were dissected and the heart, liver, spleen, lung, kidney together with tumors were used for imaging using noninvasive optical imaging system (IVIS) spectrum (n = 3 for each group). Additionally, the content of free PTX in tumors were determined using UPLC-MS-MS. In short, when the tumor volume was about 400 mm^3^, the Taxol or prodrug nanoassemblies (10 mg/kg equal to PTX) were injected into the tain vein (n = 3 for each group). At 4, 12 and 24 h after injection, the tumors were excised and weighed for content analysis.

### *In vivo* Antitumor Efficacy

The hair on the right back of the female BALB/c mice was removed with a razor beforehand, and then CT26 cells (1 × 10^6^) were seeded on the right back via subcutaneous injection to establish a CT26 tumor model. When the tumor volume grew up to 100 mm^3^, mice were indiscriminately classified into 5 groups (n = 5): untreated control (Saline), free PTX (Taxol), Abraxane, C_8_-SS-PTX NPs and F_8_-SS-PTX NPs. Then, these formulations were administrated via tail vein (8 mg/kg of PTX) for five injections every second day. The Length (L) and the width (W) of the tumor were determined daily with a caliper, and body weight were certified daily. And the tumor size was calculated as: Tumor (mm^3^) = L × W × W × 0.5. At the last day of observation, the tumors were weighed. The tumor burden was calculated as: Tumor burden (%) = (W_tumor_/W_mice_) × 100. Centrifugation was used to gain the serum for the analysis of hepatic and renal function, and the blood samples were used for blood routine examination. Moreover, the heart, liver, spleen, lung, kidney and tumor were dissected for H&E staining to evaluate physiological changes of main organs and tumors. Using TUNEL assay to evaluate the apoptosis of tumor according to the manufacturer instruction. Ki67 immunofluorescence staining was utilized to test the proliferation of tumor. The TUNEL-positive cells (green) and DAPI-positive cells (blue) were quantified by Image J, and the ratio is the relative percentage of apoptotic cells. Similarly, using Image J to quantify the fluorescent area of Ki67-positive cells (red) and the DAPI-positive cells (blue), and the ratio is the relative percentage of proliferative cells.

### Statistical Analysis

The data were performed as mean value ± standard deviation. Statistical comparisons between groups were analyzed using Student's t-test (two-tailed). Statistical significance was considered at *P < 0.05, **P < 0.01, ***P < 0.001 and ****P < 0.0001.

## Results and Discussion

### Synthesis of PTX-Perfluorooctanol and PTX-Octanol Prodrugs

The synthetic routes for PTX-Perfluorooctanol and PTX-Octanol prodrugs were shown in Figure S1. The corresponding prodrugs were named as F_8_-SS-PTX and C_8_-SS-PTX, respectively. As displayed in Figure S2 and S3, the results of MS and ^1^H NMR showed that the prodrugs were successfully synthesized. The purities of the prodrugs were over 99% (Figure S4).

### Preparation and Characterization of Prodrug Nanoassemblies

**Figure 2:**
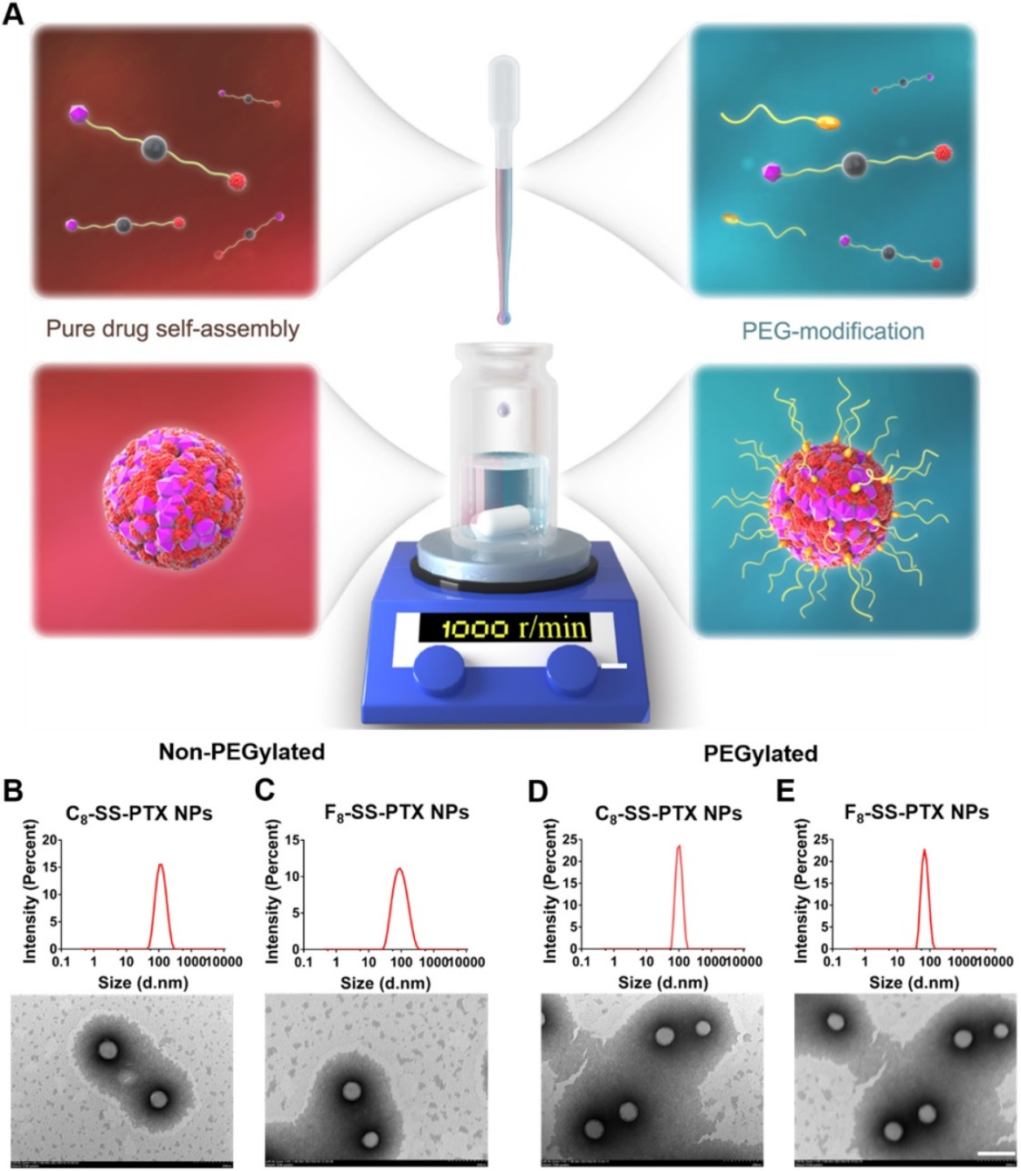
** (A)** Preparation of prodrug NPs by one-step nanoprecipitation method. Particle size distribution and TEM images of the non-PEGylated prodrug nanoassemblies for **(B)** C_8_-SS-PTX NPs, **(C)** F_8_-SS-PTX NPs. Particle size distribution and TEM images of the PEGylated prodrug nanoassemblies for **(D)** C_8_-SS-PTX NPs, **(E)** F_8_-SS-PTX NPs. Scale bar represents 200 nm.

**Figure 3:**
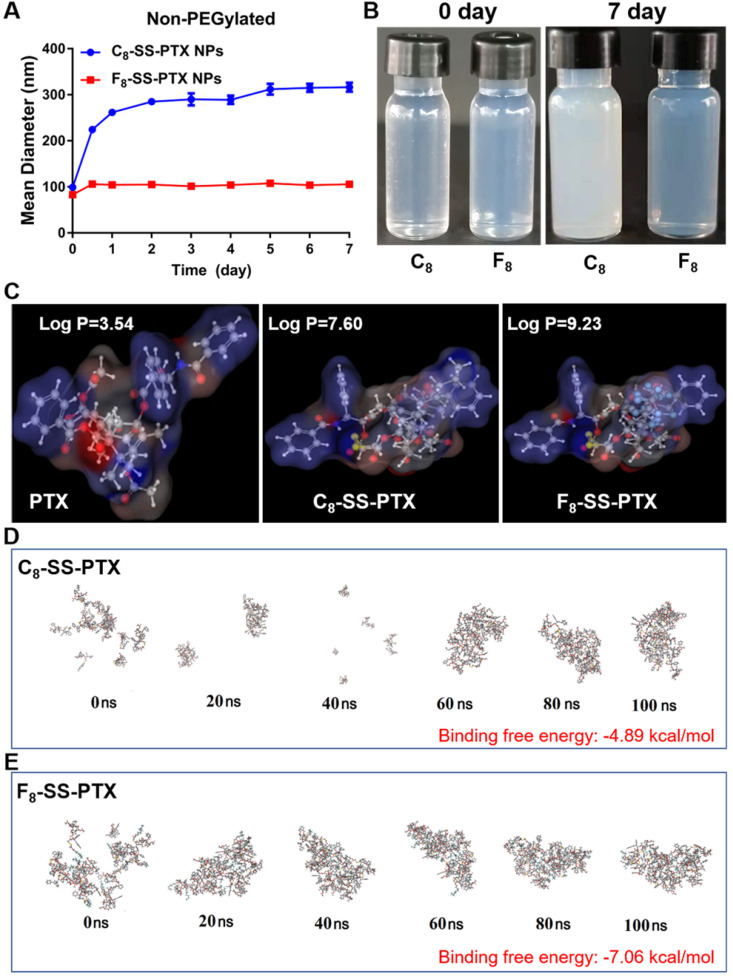
** (A)** Colloidal stability of non-PEGylated prodrug nanoassemblies after being stored for 7 d at room temperature. **(B)** The images of prodrug nanoassemblies at 0 day and 7 d. **(C)** The Log P of PTX, C_8_-SS-PTX and F_8_-SS-PTX. Assembly process of C_8_-SS-PTX **(D)** and F_8_-SS-PTX **(E)** from 0 ns to 100 ns.

Firstly, we compared the self-assembling performance of the two prodrugs without the addition of any surfactants. The one-step nanoprecipitation method was applied to prepare the prodrug nanoassemblies (Figure 2A). As displayed in Figure 2B-C and Table S1, F_8_-SS-PTX and C_8_-SS-PTX could spontaneously assemble into uniform NPs in aqueous solution. The diameter of non-PEGylated F_8_-SS-PTX NPs was smaller than that of non-PEGylated C_8_-SS-PTX NPs, suggesting that the nanostructure of F_8_-SS-PTX NPs was more compact than that of C_8_-SS-PTX NPs. Further, the colloidal stability of the two prodrug nanoassemblies was tested at room temperature. As depicted in Figure 3A, the particle size of non-PEGylated C_8_-SS-PTX NPs increased significantly, while non-PEGylated F_8_-SS-PTX NPs showed excellent colloidal stability, with almost no changes in particle size within 7 d. As seen from Figure 3B, when stored at room temperature for 7 d, C_8_-SS-PTX NPs became turbid, while F_8_-SS-PTX NPs had basically no change.

The only difference between the two prodrug nanoassemblies was the substitution of fluorine atoms. It has been reported that fluoroalkylation could facilitate the self-assembly of amphiphilic polymer due to the unique fluorine effect, including fluoro-mediated hydrophobic forces and halogen bonds 19. Therefore, the oil-water partition coefficients (Log P) of the two prodrugs were calculated by MarvinSketch, as the hydrophobic force drives the self-assembly process. As shown in Figure 3C, the Log P of PTX, C_8_-SS-PTX and F_8_-SS-PTX were 3.54, 7.60 and 9.23, respectively. The highly hydrophobic F_8_-SS-PTX had strong intermolecular forces, thereby promoting the self-assembly of prodrug molecules. In addition, the self-assembly mechanisms were explored by molecular dynamics simulation. As shown in Figure S5A and S5B, multiple intermolecular interactions were involved in the self-assembly process, such as π-π stacking (pink dotted line), alkyl-π stacking (yellow dotted line), π-S stacking (red dotted line), hydrogen bond (green dotted line) and halogen bond (cyan-blue dotted line). For C_8_-SS-PTX, the alkyl-π action was the main force for its assembly, and for F_8_-SS-PTX, due to the effect of fluorine atoms, the halogen bond (C=O…F) was the main force for its assembly, as illustrated in Figure S5C and S5D. In addition, F_8_-SS-PTX possessed more intermolecular hydrogen bonds than C_8_-SS-PTX (Figure S5E), which could improve the stability of nanostructure. Furthermore, the free binding energy value of F_8_-SS-PTX NPs (-7.06 kcal/mol, Figure 3E) was much lower than that of C_8_-SS-PTX NPs (-4.89 kcal/mol, Figure 3D). In terms of the thermodynamic laws, negative values of the free binding energy indicate the favorable stability of the system, and the larger potential energy absolute values, the more stable structure would be shaped 32, 33. Moreover, in the process of molecular dynamics simulation, F_8_-SS-PTX self-assembled into stable nanostructure in about 10 ns (Figure 3E and Figure S5F), while C_8_-SS-PTX formed the similar nanostructure until 90 ns (Figure 3D and Figure S5F), demonstrating that F_8_-SS-PTX had a significantly better self-assembling ability. These findings illustrated that fluoroalkylation of the carbon chains could effectively improve the self-assembly capability of PTX-fatty alcohol prodrugs and the stability of prodrug nanoassemblies.

To improve the *in vivo* disposition fate of prodrug nanoassemblies, prodrugs were co-assembled with DSPE-PEG_2k_ to engineer a stealth PEG surface. As illustrated in Figure 2D-E and Table S2, the average diameter of the PEGylated prodrug nanoassemblies was near to 90 nm, and the zeta potential was close to -20 mV. TEM images clearly demonstrated the successful formation of spherical prodrug nanoassemblies. Since the prodrugs act as the dual roles of cargoes and carriers, the prodrug nanoassemblies exhibited extra-high drug loading efficiency (more than 50% PTX equivalent, wt.%, Table S2). The PEGylated prodrug nanoassemblies were used for the follow-up experiments.

The colloidal stability of the PEGylated formulations was evaluated in the four conditions: (i) at 4 °C; (ii) at room temperature; (iii) in PBS solution (pH 7.4) containing 10% FBS at 37 °C; and (ⅳ) in culture medium (pH 7.4 and pH 5.0) containing 10% FBS at 37 °C. As displayed in Figure S6, PEGylation did not change the stability difference among the two prodrug nanoassemblies. Compared with C_8_-SS-PTX NPs, F_8_-SS-PTX NPs still exhibited better colloidal stability, which may be beneficial for the *in vivo* pharmacokinetic behavior and tumor accumulation.

### Redox-Responsive Drug Release

**Figure 4:**
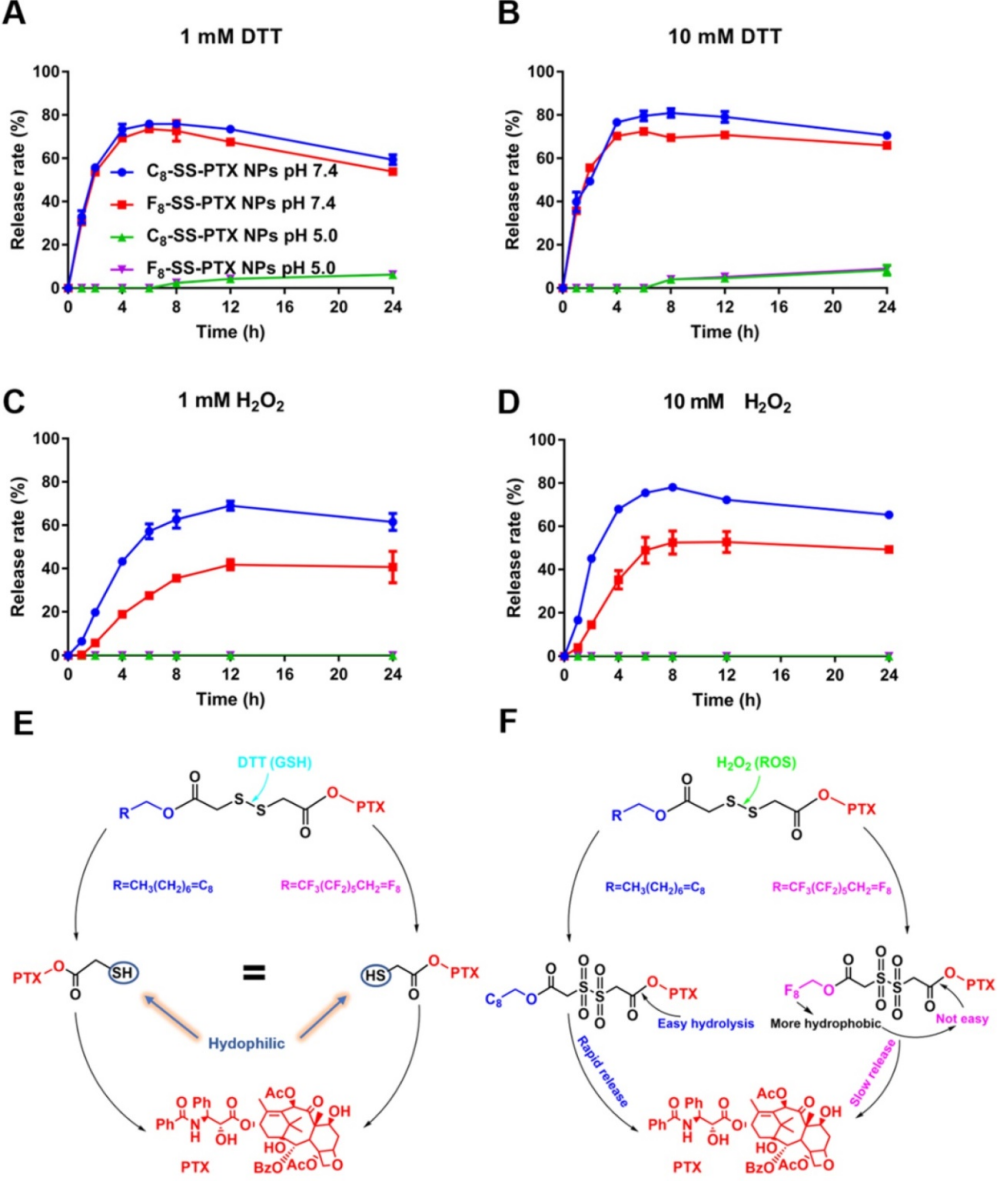
*** In vitro* redox-responsive drug release. (A)** 1 mM DTT; **(B)** 10 mM DTT; **(C)** 1 mM H_2_O_2_; **(D)** 10 mM H_2_O_2_. Data are presented as the mean ± SD (n = 3). The mechanism of reduction-triggered **(E)** and oxidation-triggered **(F)** drug release.

The released active PTX was confirmed by MS spectrum (Figure S7). As depicted in Figure 4, under acidic conditions (pH 5.0), there was almost no release of paclitaxel, due to the inefficient hydrolysis of ester in acid conditions 30. Under neutral condition, no matter concentration of DTT was low (1 mM) or high (10 mM), the drug release rate was very fast and basically the same. Moreover, the release rates of C_8_-SS-PTX NPs and F_8_-SS-PTX NPs were almost equal, which was consistent with the reduction-triggered mechanism of disulfide. The reduction-triggered mechanism of prodrug nanoassemblies was displayed in Figure 4E 16. The disulfide bond was transformed into hydrophilic thiol intermediate (PTX-SH) under the attack of DTT, which could promote the breakage of the PTX-linked ester bond, then releasing the free PTX. Because the hydrophilic thiol groups generated by the two prodrugs were the same, the release rate of the two prodrugs in the reduction environment was almost equivalent. The molecule weights of the intermediate (PTX-SH) were confirmed by mass spectrometry (Figure S8).

The two prodrug nanoassemblies also exhibited H_2_O_2_-responsive drug release, and the release rate was ranked as C_8_-SS-PTX NPs > F_8_-SS-PTX NPs (Figure 4C-D). The oxidization-responsive mechanism of the disulfide bond had been clearly clarified, and the sulfur atoms were oxidized by H_2_O_2_ to generate hydrophilic sulfoxide or sulphone 16, thereby facilitating the release of free PTX (Figure 4F). Because F_8_-SS-PTX was more hydrophobic than C_8_-SS-PTX, it had stronger ability to prevent water from attacking the ester bond. As a result, the release rate of F_8_-SS-PTX in the oxidation condition was slower than C_8_-SS-PTX. We then confirmed the molecule weights of the intermediate using mass spectrometry. The mass spectra of C_8_-SS-PTX (1185) and F_8_-SS-PTX (1402) indicated the formation of corresponding sulfone products (Figure S9).

Moreover, we further analyzed the *in vitro* hydrolysis of the prodrug at pH 7.4 and pH 5.0. As shown in Figure S10, under the condition of 1 mM DTT, pH had a great influence on the hydrolysis of prodrug nanoassemblies. Acidic condition inhibited the hydrolysis of prodrug nanoassemblies, which had been reported by the previous study 30. Briefly, the disulfide was cleaved by the deprotonated thiol of DTT. Therefore, the reaction would happen easily at a higher pH. However, under the condition of 10 mM DTT, both the prodrug nanoassemblies were hydrolyzed quickly no matter the pH was 7.4 or 5.0. It was because the high concentration of DTT accelerated the reaction with the disulfide bond. Moreover, under the condition of 1 mM DTT and 10 mM DTT (pH 7.4), both the two prodrug nanoassemblies were hydrolyzed within 1 h, illustrating the disulfide was very sensitive to the reductive environment, which also was consistent with the result of release behavior. In contrast, the degradation rate of the two prodrug nanoassemblies under oxidative conditions was slower. Under 10 mM H_2_O_2_ (pH 7.4), the prodrug nanoassemblies were not completely degraded until 8 h. In addition, the degradation rates of C_8_-SS-PTX NPs and F_8_-SS-PTX NPs were basically the same, further demonstrating that the slower drug release rate of F_8_-SS-PTX NPs under oxidative conditions was due to its stronger hydrophobicity which prevented water from attacking the ester bond. pH also exerted a great impact on the oxidation of disulfide bonds, under acidic conditions (pH 5.0), the hydrolysis of the prodrug was severely hindered, because the hydrolysis of the ester bond was hindered under acidic conditions, leading to accumulation of oxidation intermediates and hindering the further progress of the oxidation reaction.

In addition, the prodrug nanoassemblies incubated with 10 mM DTT (pH 7.4 and pH 5.0) or 10 mM H_2_O_2_ (pH 7.4 and pH 5.0) for 24 h were observed by TEM. As illustrated in Figure S11, under the condition of 10 mM DTT, both F_8_-SS-PTX NPs and C_8_-SS-PTX NPs were disassembled, suggesting the reduction-responsive hydrolysis of prodrugs. Under the condition of 10 mM H_2_O_2_, no presence of intact nanoparticles was observed at pH 7.4. In contrast, a large number of nanoparticles were observed at pH 5.0. These results suggested that under acidic conditions (pH 5.0), the oxidation of prodrug nanoassemblies was hindered, consisting with the result of the *in vitro* release.

### Cellular Uptake

**Figure 5:**
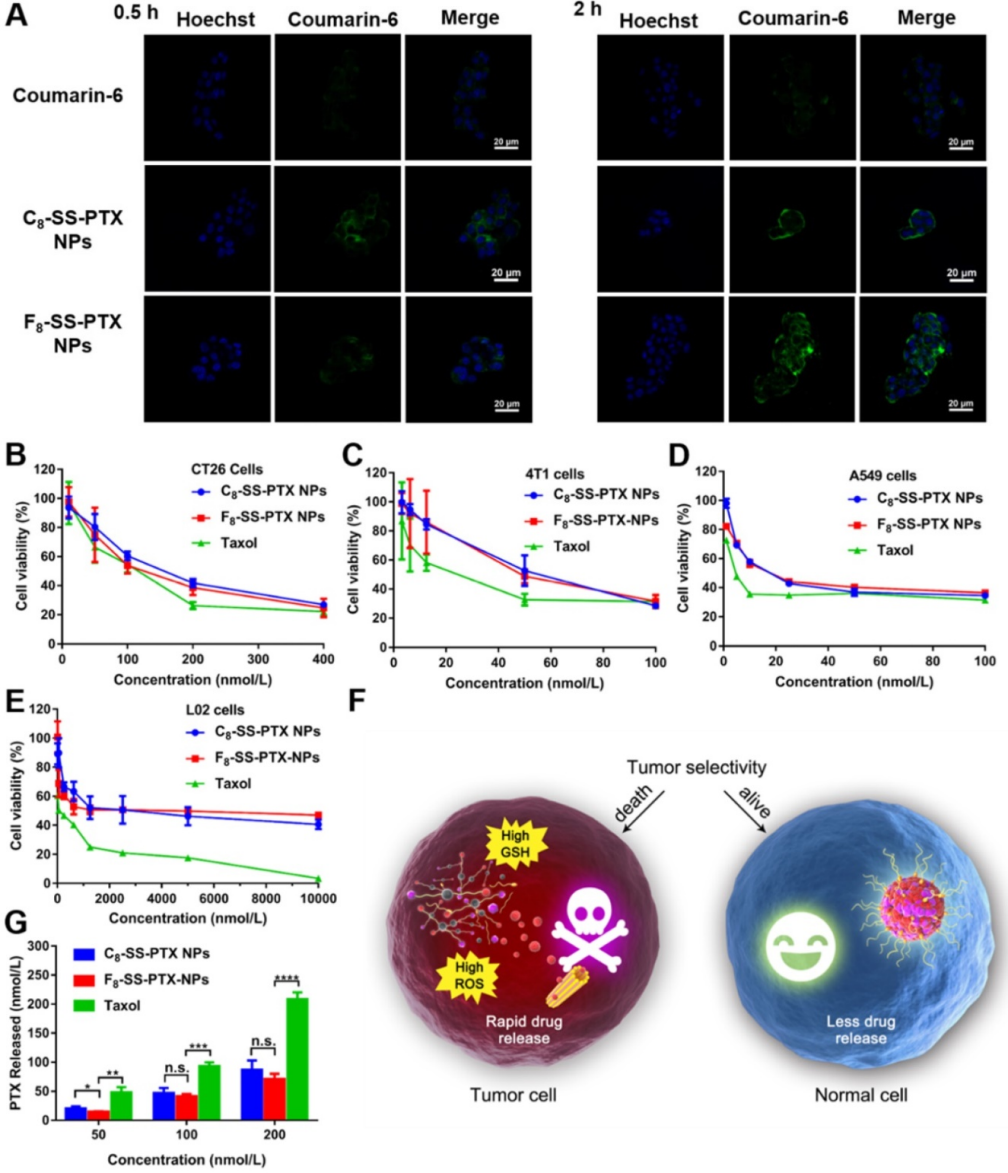
** (A)** CLSM images of CT26 cells incubated with free coumarin-6 or coumarin-6-labeled prodrug nanoassemblies for 0.5 h and 2 h. Scale bar represents 20 µm. Cell viability treated with various concentrations of Taxol and prodrug nanoassemblies. **(B)** CT26 cells,** (C)** 4T1 cells, **(D)** A549 cells and **(E)** L02 cells. **(F)** The selective bioactivation of prodrug nanoassemblies in tumor cells and normal cells. **(G)** Free PTX released from prodrug nanoassemblies after incubation with CT26 cells for 48 h. Data are presented as mean ± SD (n = 3).

As shown in Figure 5A, compared with free coumarin-6, cells treated with coumarin-6-marked prodrug nanoassemblies displayed notably higher intracellular fluorescence intensity, which suggested the uptake efficiency of nanoassemblies was better than that of free drug solution. Furthermore, these prodrug nanoassemblies showed the comparable cellular uptake efficiency. In addition, we also measured the intracellular fluorescence intensity by flow cytometry and extended the incubation time. As shown in Figure S12, when incubating for a long time, the uptake efficiency of prodrug nanoassemblies was still significantly higher than that of coumarin-6 solution. Furthermore, there was no significant difference between C_8_-SS-PTX NPs and F_8_-SS-PTX NPs due to their very similar surface properties (Table S2) 17, 31.

### Cytotoxicity and Intracellular Drug Release

The cytotoxicity of the prodrug nanoassemblies on CT26 cells, 4T1 cells, A549 cells and L02 cells was investigated utilizing the MTT method. The half maximal inhibitory concentration (IC_50_) values were displayed in Table S3. Both the prodrug nanoassemblies showed lower cytotoxicity than Taxol, as a result of the delayed release of active PTX. The two prodrug nanoassemblies exhibited comparable cytotoxicity (Figure 5B-E). It has been reported that the cytotoxicity depended on the amount of intracellular released active parent drug 17, 30, 31. Then, the amount of free PTX released from the prodrug nanoassemblies was determined using CT26 cells as the model. As shown in Figure 5G, there was no obvious difference in the amount of free PTX released from the two prodrug nanoassemblies in CT26 cells, providing the foundation for the result of cytotoxicity.

Moreover, the toxicity of prodrug nanoassemblies to normal cells was much lower than that of Taxol. The tumor selective index (SI) was calculated and displayed in Table S4. The SI values of prodrug nanoassemblies were much higher than that of Taxol, indicating that the prodrug nanoassemblies could kill tumor cells selectively owing to the redox-triggered intracellular drug release (Figure 5F), thereby alleviating the systemic toxicity of PTX 34.

### AM/PI staining and cell apoptosis

The results of AM/PI staining were shown in Figure S13A, the cells treated with blank medium grown well, while significant damage was observed for the cells treated with C_8_-SS-PTX NPs and F_8_-SS-PTX NPs. In comparison, a mass of dead cells was observed when treated with Taxol. In addition, cell apoptosis by staining with Annexin-V FITC and propidium iodide (PI) was also determined using flow cytometric measurement. As shown in Figure S13B, the apoptosis rate was 2.57% in the control group. In comparison, Taxol induced the highest apoptosis rate (23.37%). C_8_-SS-PTX NPs induced 11.46% apoptosis rate, which was basically equivalent to that induced by F_8_-SS-PTX NPs (12.63%).

### *In vitro* microtubule polymerization assay

As illustrated in Figure S14, after CT26 cells were treated with prodrug nanoassemblies for 48 h, the microtubules and nuclei were stained with α-tubulin antibody (red) and Hoechst 33342 (blue), respectively. The red fluorescence intensity in C_8_-SS-PTX NPs and F_8_-SS-PTX NPs-treated cells wasn't obviously different, higher than that of the control group. The results illustrated that C_8_-SS-PTX NPs and F_8_-SS-PTX NPs could release equivalent PTX in tumor cells, thereby inhibiting the depolymerization of microtubules. In addition, the red fluorescence intensity of Taxol-treated cells was strongest, consistent with the results of cytotoxicity (Figure 5B-D).

### Hemolysis assay

In order to preliminarily evaluate the safety of the prodrug nanoassemblies, we conducted a hemolysis test. As shown in Figure S15, the C_8_-SS-PTX NPs and F_8_-SS-PTX NPs did not cause hemolysis, and there was no significant difference from the saline group, which preliminarily showed that the prodrug nanoassemblies were safe and could be used for intravenous injection.

### *In vivo* Pharmacokinetics and Biodistribution

**Figure 6:**
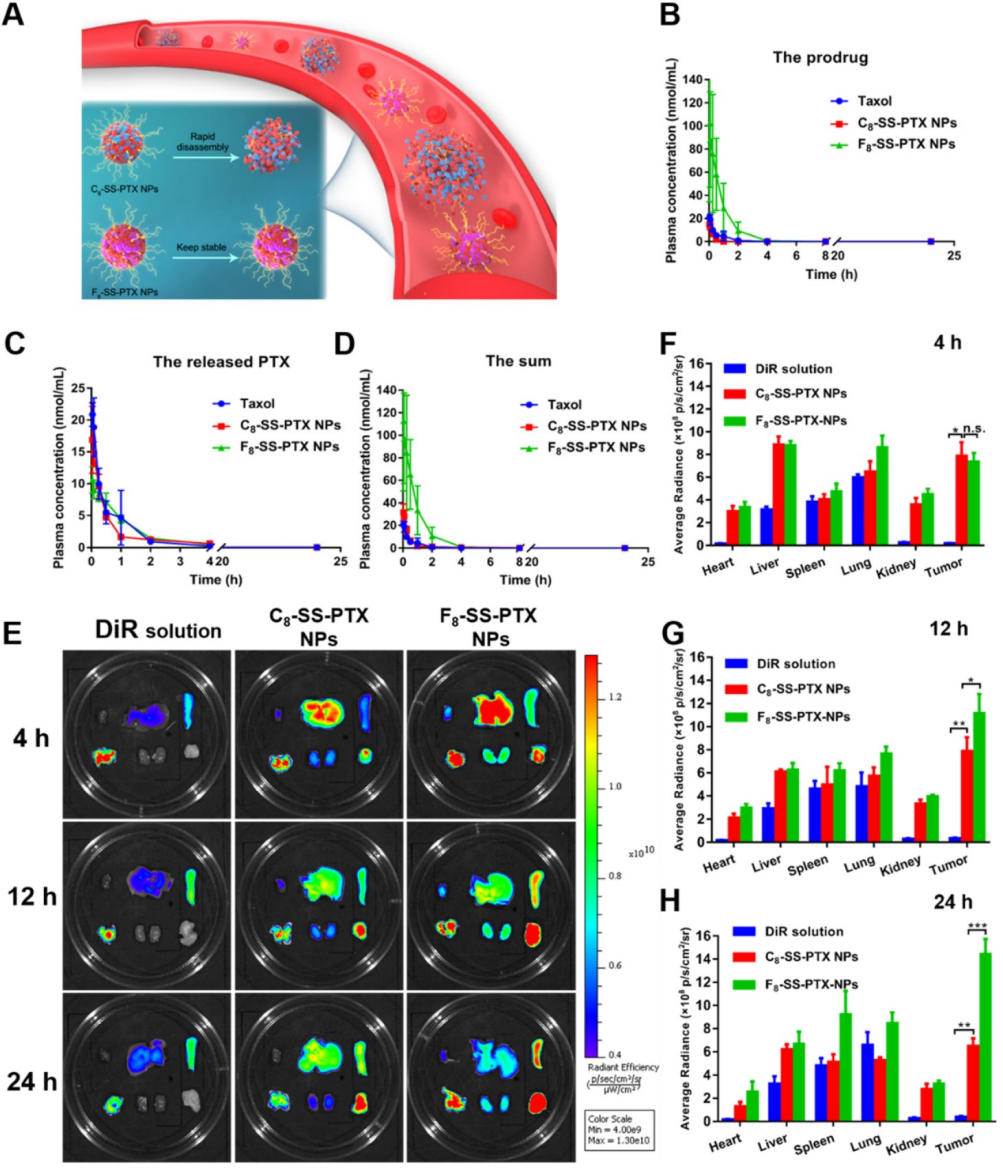
** (A)** Colloidal stability of prodrug nanoassemblies correlated with their blood circulation. Pharmacokinetic profiles of prodrug nanoassemblies (n = 5). Molar concentration-time curves of the prodrugs **(B)**, the released PTX **(C)** and the sum of them **(D)**. *Ex vivo* biodistribution of DiR-labeled prodrug nanoassemblies (n = 3). **(E)** Fluorescent imaging at different time. Quantitative results of relative organ and tumor accumulation at 4 h **(F)**, 12 h **(G)** and 24 h **(H)**.

The pharmacokinetic profiles were displayed in Figure 6B-D, and the pharmacokinetic parameters were shown in Table S5. PTX in Taxol was quickly cleared from the blood. By contrast, prodrug nanoassemblies significantly improved the *in vivo* fate of PTX. The AUC of total PTX in the F_8_-SS-PTX NPs and C_8_-SS-PTX NPs were approximately 7.7- and 1.3- times higher than those of Taxol, respectively. The F_8_-SS-PTX NPs possessed a much better blood circulation than the C_8_-SS-PTX NPs, possibly as a result of the improved colloidal stability contributed by the fluoroalkylation (Figure 6A). These results demonstrated that the fluoroalkyl tail in prodrug structures had a significant effect on the pharmacokinetic behavior. We further investigated the chemical stability of the prodrug nanoassemblies in the fresh rat plasma. As shown in Figure S16, C_8_-SS-PTX NPs were extremely unstable in plasma and degraded rapidly, consistent with the result of the *in vivo* pharmacokinetics behavior. In comparison, the stability of F_8_-SS-PTX NPs was much higher than C_8_-SS-PTX NPs.

The improved pharmacokinetic behavior could facilitate prodrug nanoassemblies accumulation in tumor. Therefore, the IVIS spectrum was used to study the biodistribution of prodrug nanoassemblies labeled by DiR on BALB/c mice bearing CT26 tumor. As displayed in Figure 6E-H, DiR solution mainly accumulated in lung, almost no distribution in tumor. By contrast, prodrug nanoassemblies exhibited notably higher fluorescent intensity in tumor, which would be attributed to the EPR effect and the improved pharmacokinetic behavior. Moreover, the accumulation of prodrug nanoassemblies in tumor were well in accordance with their self-assembling stability and pharmacokinetic behavior as described above. F_8_-SS-PTX NPs, with enhanced self-assembling stability and better pharmacokinetic behavior, showed higher accumulation in tumor than C_8_-SS-PTX NPs. Furthermore, the amount of PTX in tumor were measured using UPLC-MS-MS, the determinative factor of the final antitumor effect. As shown in Figure S17, the content of free PTX in the tumor tissues of F_8_-SS-PTX NPs group was significantly higher than that of C_8_-SS-PTX NPs group. These results indicated that fluoroalkylation could ameliorate the antitumor effect of prodrug nanoassemblies.

### *In vivo* Antitumor Effect

**Figure 7:**
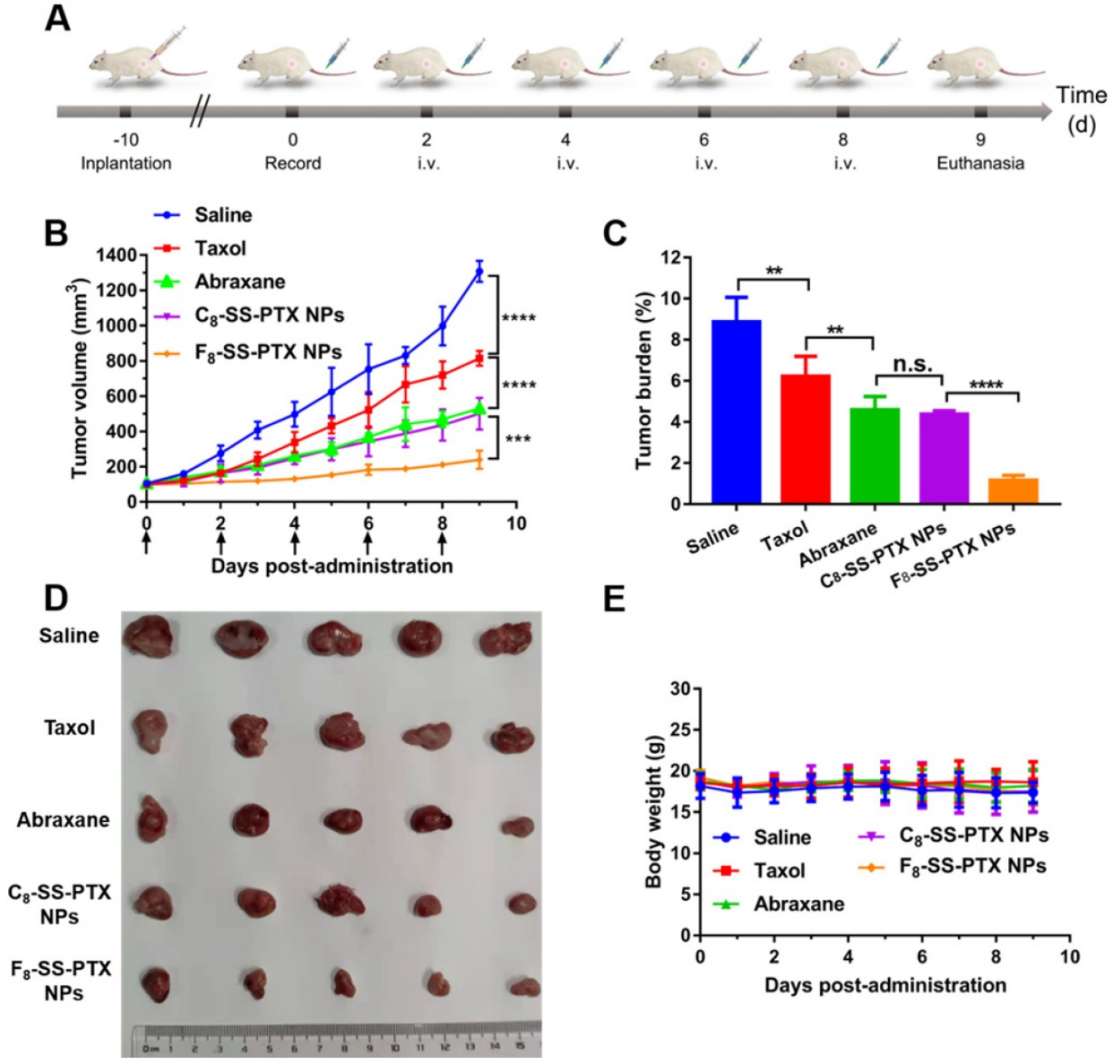
** (A)** Schedule for *in vivo* antitumor therapy. *In vivo* antitumor activity of prodrug nanoassemblies against CT26 xenograft tumors (n = 5). **(B)** Tumor growth curves after treated with different formulations. **(C)** Tumor burden. **(D)** Pictures of tumors after last treatment. **(E)** Body weight changes. The data are presented as means ± SD. **P < 0.01, ***P < 0.001 and ****P < 0.0001 by two-tailed Student's t test.

Then, the impact of fluoroalkylation on the antitumor effect of prodrug nanoassemblies was investigated using BALB/c mice bearing CT26 tumor (Figure 7A). As illustrated in Figure 7B-D, there was no significant difference between C_8_-SS-PTX NPs and Abraxane, the commercial PTX albumin NPs. In comparison, F_8_-SS-PTX NPs exhibited the best antitumor effect with the smallest tumor volume and tumor burden. Then, the cellular apoptosis of tumor was assessed by TUNEL assay. As shown in Figure S18A-B, compared with Taxol, the prodrug nanoassemblies and Abraxane caused a large number of tumor apoptosis, especially F_8_-SS-PTX NPs. Further, the Ki67 assay was used to evaluate the cellular proliferation of tumor tissues (Figure S18C-D). Compared with other groups, F_8_-SS-PTX NPs-treated groups showed the smallest proliferation in tumor tissues, suggesting the best antitumor efficacy. The improved antitumor activity of F_8_-SS-PTX NPs should be due to the multiple therapeutic superiorities, including better colloidal stability, improved pharmacokinetic behavior and higher tumor accumulation. The application of fluoroalkyl tail improved the self-assembly performance of prodrugs and made the prodrug nanoassemblies more stable, thereby prolonging the circulation time and enabling more prodrug nanoassemblies to reach the tumor.

No significant changes in body weight (Figure 7E), complete blood test (Figure S19A) and hepatorenal function (Figure S19B) were found in all groups. H&E staining results also explained that all formulations have no obvious damage to the main organs, as displayed in Figure S20. All of the results demonstrated that the PTX prodrug nanoassemblies exerted excellent therapeutic effects but negligible toxicity.

## Conclusions

To investigate the effect of fluoroalkylation on the self-assembly performance and drug delivery efficiency of prodrug nanoassemblies, we herein synthesized two prodrugs by conjugating PTX with perfluorooctanol or octanol via disulfide bond. The two hydrophobic prodrugs could self-assemble into uniform nanoassemblies with extra-high drug loading (over 50%). Firstly, we found that replacing hydrogen atoms with fluorine atoms in carbon chains greatly improved the self-assembling capability and colloidal stability of prodrug nanoassemblies due to the fluoro-mediated hydrophobic characteristic and halogen bond. The stable F_8_-SS-PTX NPs, with improved pharmacokinetic behavior and improved tumor accumulation, exhibited much better antitumor efficacy than C_8_-SS-PTX NPs and commercial Abraxane. Our findings clarified the impact of fluoroalkylation on the self-assembling characteristics and drug delivery effectiveness of prodrug nanoassemblies, and provided a new strategy for the rational design of advanced prodrug-based nanomedicines.

## Supplementary Material

Supplementary figures and tables.

## Abbreviations

NPs: nanoparticles
EDCI: 1-Ethyl-3-(3-dimethyllaminopropyl) carbodiimide hydrochloride
DMAP: 4-dimethylaminopyrideine
DSPE-PEG2k: 1,2-distearoylsn-glycero-3-phosphoethanolamine-N-[methoxy(polyethyleneglycol)-2000
DiR: 1,1'-dioctadecyl-3,3,3',3'-tetramethylindotricarbocyanine iodide
HPLC: high performance liquid chromatography
PDI: polydispersion index
TEM: transmission electron microscopy
DTT: DL-Dithiothreito
DAPI: 4',6-diamidino-2-phenylindole
MTT: 3-(4,5-dimethyl-2-thiazolyl)-2,5-diphenyl-2-H-tetrazolium bromide
FBS: fetal bovine serum
CLSM: confocal laser scanning microscopy
H&E: hematoxylin and eosin
EPR: enhanced permeability and retention
DAPI: 4',6-Diamidino-2-phenylindole dihydrochloride
