# Supplementary material for: Probing the fluorination effect on the self-assembly characteristics, in vivo fate and antitumor efficacy of paclitaxel prodrug nanoassemblies
Source: Theranostics. 2021 Jul 6;11(16):7896–910. doi: 10.7150/thno.61337 (PMC8315070; doi:10.7150/thno.61337)
Supplement: Supplementary file 1 — Supplementary figures and tables. [file thnov11p7896s1.pdf]

Supplementary materials

Title

Probing the fluorination effect on the self-assembly characteristics, *in vivo* fate and antitumor efficacy of paclitaxel prodrug nanoassemblies

Authors:

Xin Wang[a,1], Bin Yang[b,1], Lingxiao Li[a], Tian Liu[a], Shiyi Zuo[a], Dongxu Chi[c], Zhonggui He[a], Bingjun Sun[a]*, Jin Sun[a]*

Affiliations:

[1]Both authors contributed equally

[a]Department of Pharmaceutics, Wuya College of Innovation, Shenyang Pharmaceutical University, Shenyang 110016, China

[b]Department of Pharmacy, China Medical University, 155 Nanjing South Street, Shenyang 110016, China

[c]School of Pharmacy, Shenyang Pharmaceutical University, Shenyang, 110016, China

***Corresponding authors:**

Bingjun Sun, Ph.D. and Jin Sun, Ph.D.

Tel: +86-24-23986321; Fax: +86-24-23986321

E-mail: sunbingjun_spy@sina.com; sunjin@syphu.edu.cn

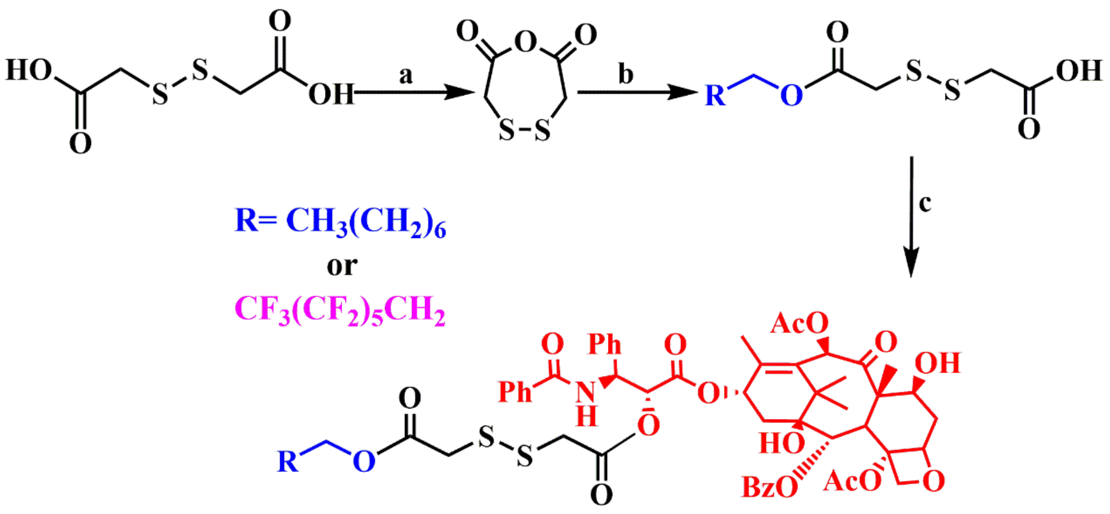

Figure S1. The synthetic route of disulfide bond-bridged PTX-Perfluorooctanol and PTX-Octanol prodrugs. (a) acetic anhydride, 25 °C; (b) perfluorooctanol or octanol, DMAP, 25 °C; (c) EDCI, DMAP, 0 °C; PTX, 25 °C.

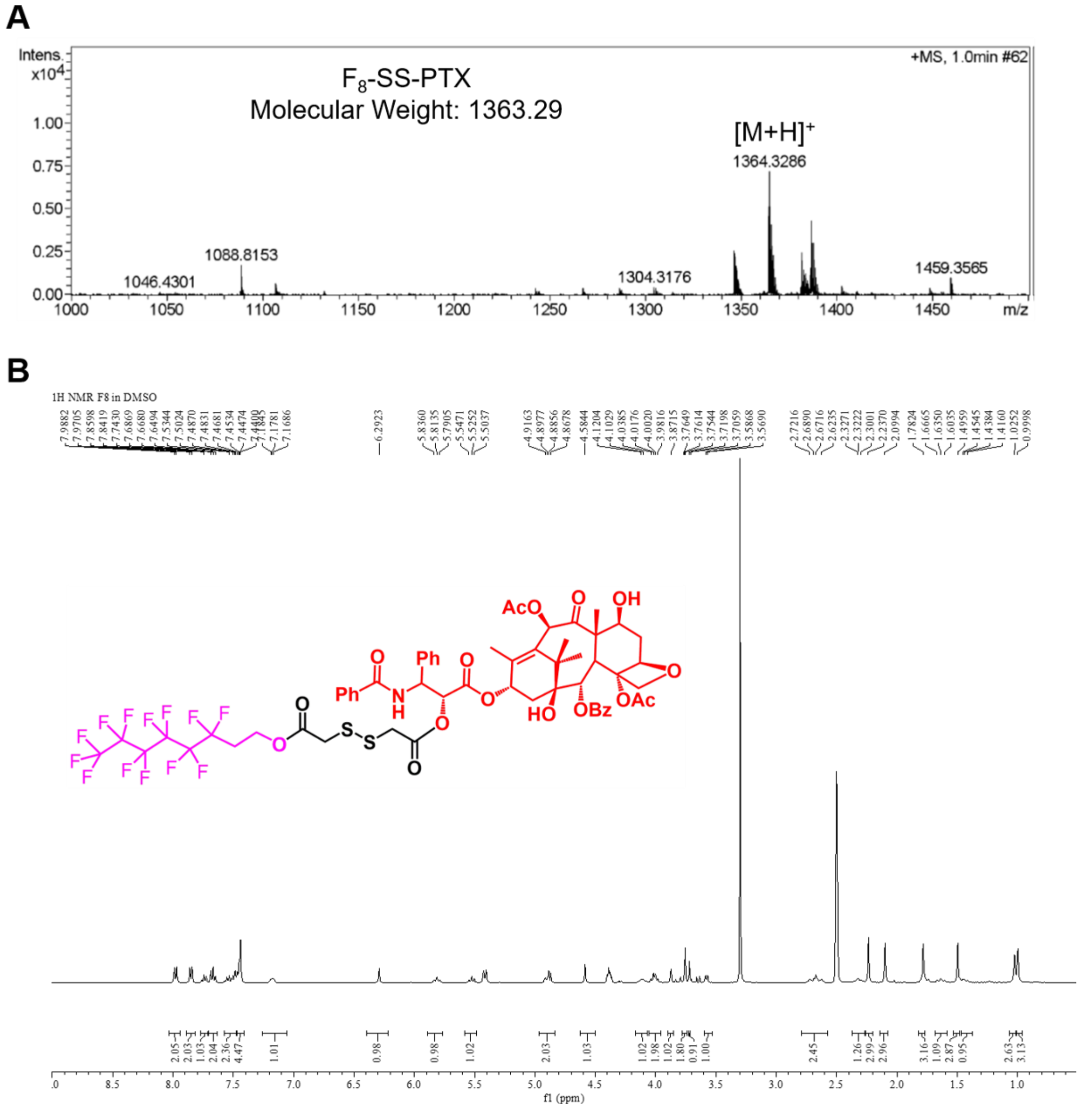

Figure S2. Characterizations of F8-SS-PTX. (A) Mass spectrum of F8-SS-PTX. (B) ^{1}H NMR spectrum of F8-SS-PTX.

^{1}H NMR (400 MHz, DMSO-d_6) δ 7.979 (d, J = 7.1 Hz, Ar-H, 2H), 7.851 (d, J = 7.2 Hz, Ar-H, 2H), 7.743 (t, J = 7.3 Hz, Ar-H, 1H), 7.668-7.058 (Ar-H, 10H), 6.292 (s, 10-CH, 1H), 5.813 (t, J = 9.1 Hz, 13-CH, 1H), 5.525 (t, J = 8.7 Hz, 3'-CH, 1H), 4.892 (dd, J = 12.1, 7.3 Hz, 2'-CH, 1H), 4.584 (s, 5-CH, 1H), 4.112 (d, J = 7.0 Hz, 7-CH, 1H), 4.010 (q, J = 8.3 Hz, 20-CH_2, 2H), 3.871-3.720 (CH_2SSCH_2,4H), 3.578 (d, J = 7.1 Hz, 3-CH, 1H), 2.697-2.311 (F8-CH_2, 4H), 2.237 (S, 4-$COCH_3$, 3H), 2.099 (S, 10-$COCH_3$, 3H),

1.782 (s, 18-CH_3, 3H), 1.635 (t, J = 12.6 Hz, 6-CH_2-αH, 1H), 1.496 (s, 19-CH_3, 3H), 1.479(m, 6-CH_2-βH, 1H), 1.025 (s, 17-CH_3, 3H), 0.998 (s, 16-CH_3, 3H).

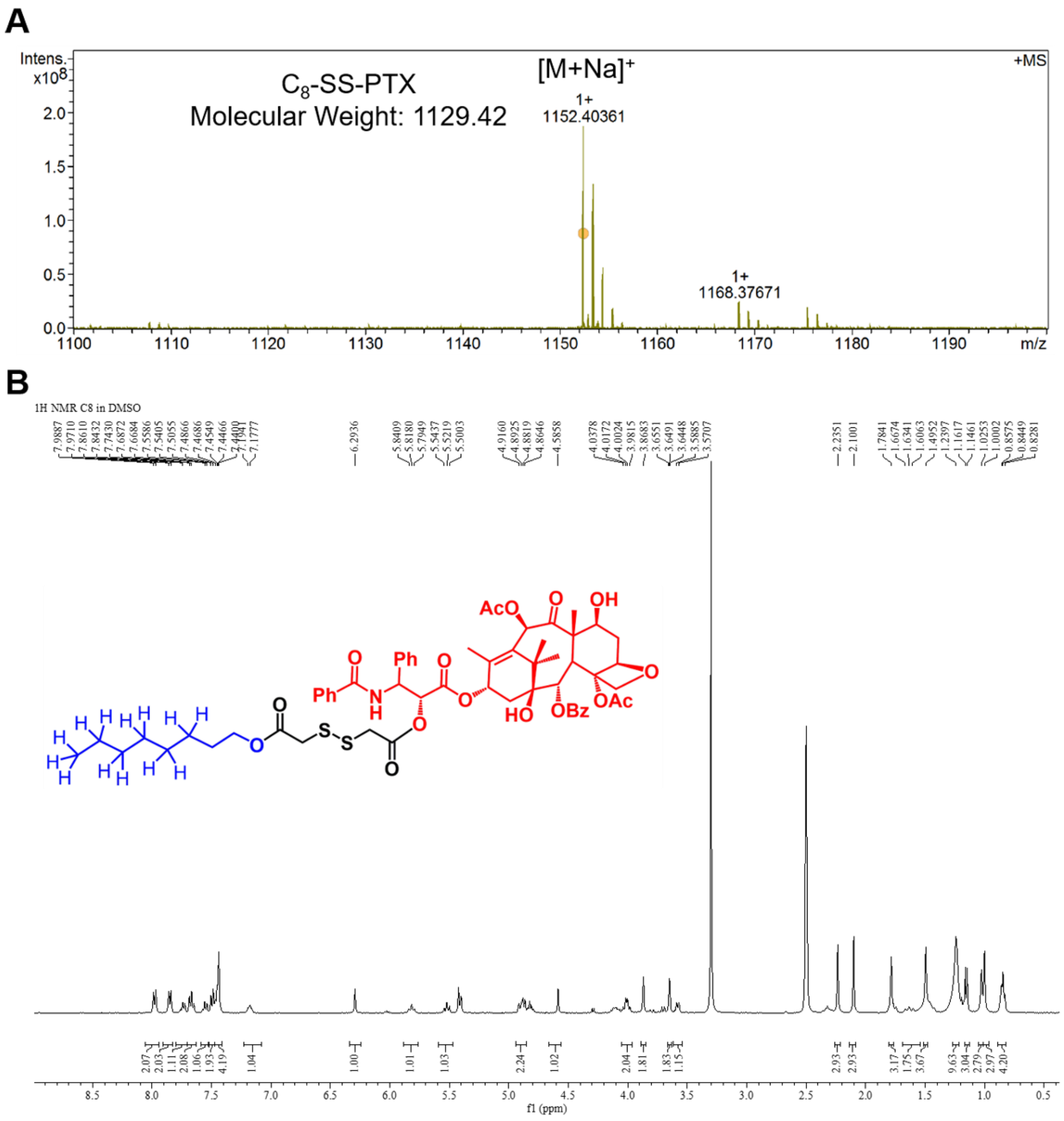

Figure S3. Characterizations of C_8-SS-PTX. (A) Mass spectrum of C_8-SS-PTX. (B) ^{1}H NMR spectrum of C_8-SS-PTX.

^{1}H NMR (400 MHz, DMSO-d6) δ 7.980 (d, J = 7.1 Hz, Ar-H, 2H), 7.852 (d, J = 7.1 Hz, Ar-H, 2H), 7.743 (t, J = 7.3 Hz, Ar-H, 1H), 7.668-7.186 (Ar-H, 10H), 6.294 (s, 10-CH, 1H), 5.818 (t, J = 9.2 Hz, 13-CH, 1H), 5.522 (t, J = 8.7 Hz, 3'-CH, 1H), 4.818 (q, J =6.4 Hz, 2'-CH, 1H), 4.586 (s, 5-CH, 1H), 4.096 (m, 7-CH, 1H), 4.010 (q, J = 8.3 Hz, 20-CH2, 2H), 3.868-3.650 (CH2SSCH2, 4H), 3.580 (d, J = 7.1 Hz, 3-CH, 1H), 2.235 (s, 4-COCH3, 3H), 2.100 (s, 10-COCH3, 3H), 1.784 (s, 18-CH3, 3H), 1.692-1.538 (m, 6-

CH2, 2H), 1.495 (s, 19-CH3, 3H), 1.240 (s, C8-CH2, 10H), 1.154 (d, J=6.2 Hz, C8-CH3, 3H), 1.025 (s, 17-CH3, 3H), 1.000 (s, 16-CH3, 3H), 0.844 (t, J = 5.9 Hz, C8-CH2, 4H).

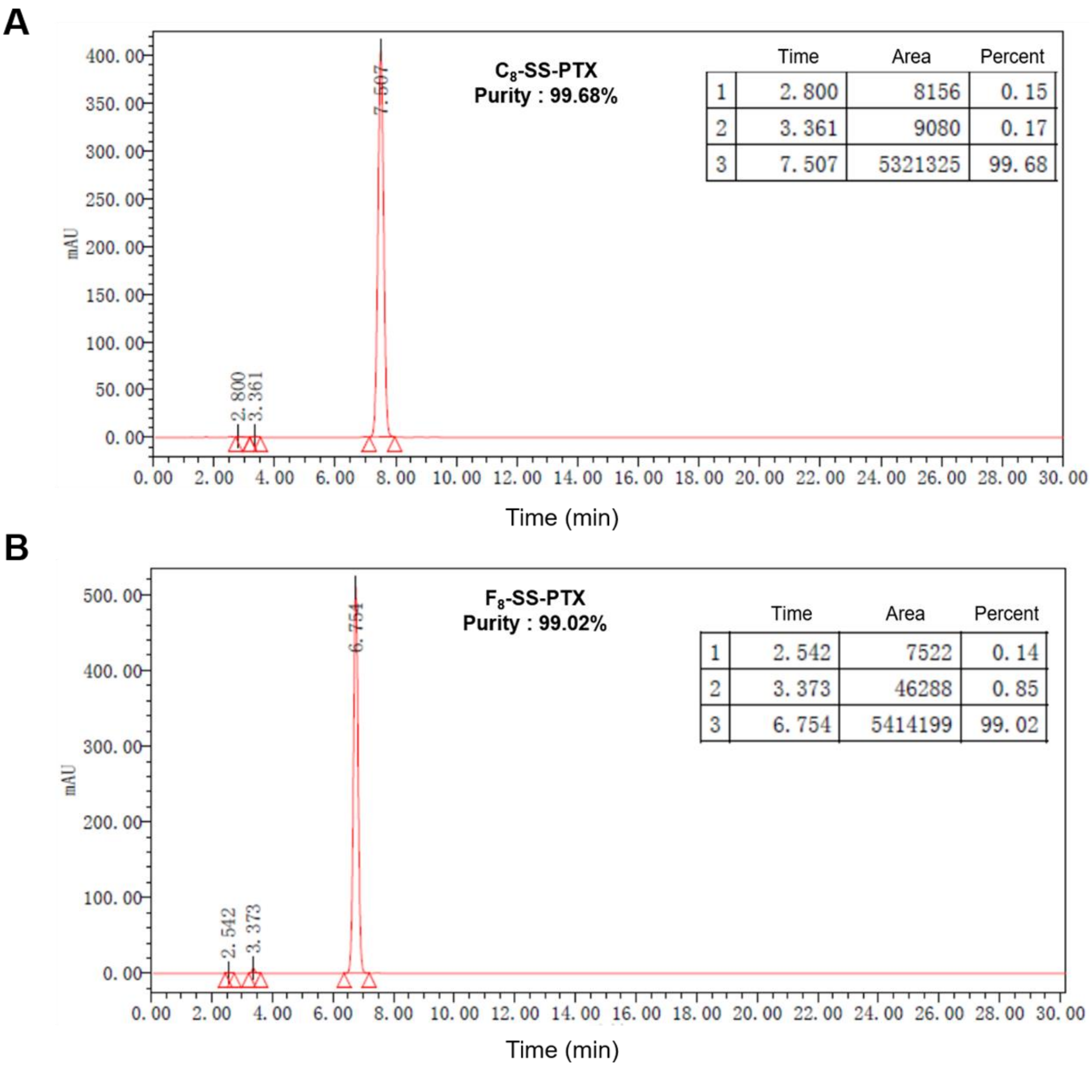

Figure S4. The purity of (A) C_8-SS-PTX and (B) F_8-SS-PTX.

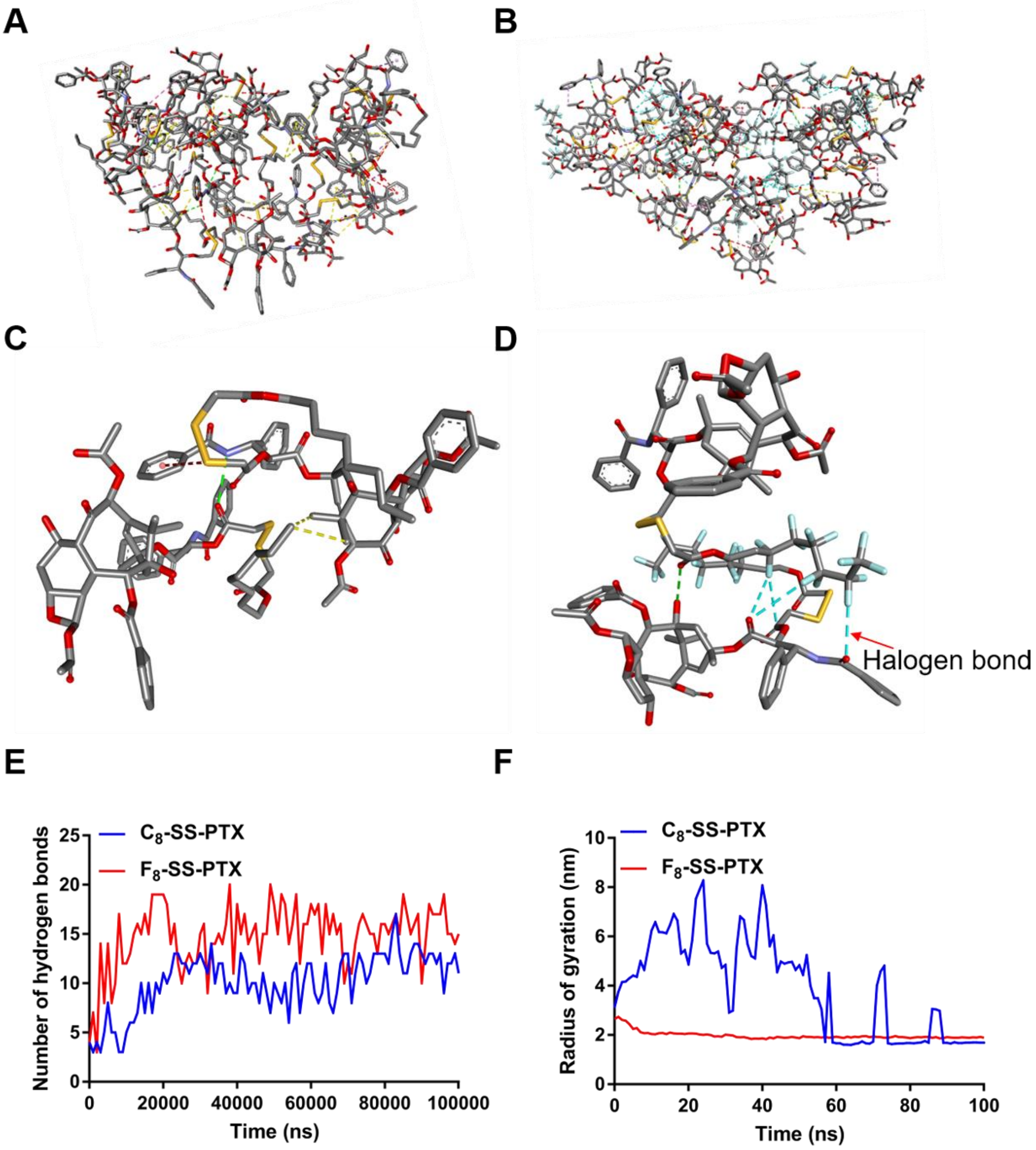

Figure S5. Molecular dynamics simulations of prodrug nanoassemblies. The molecular interaction of C_8-SS-PTX (A) and F_8-SS-PTX (B). Partial enlarged view of structural details of C_8-SS-PTX (C) and F_8-SS-PTX (D). Pink dotted line indicates π-π stacking, yellow dotted line indicates alkyl-π stacking, red dotted line indicates π-S stacking, green dotted line indicates hydrogen bond and cyan-blue dotted line indicates halogen bond. The number of hydrogen bond (E) and gyration radius (F).

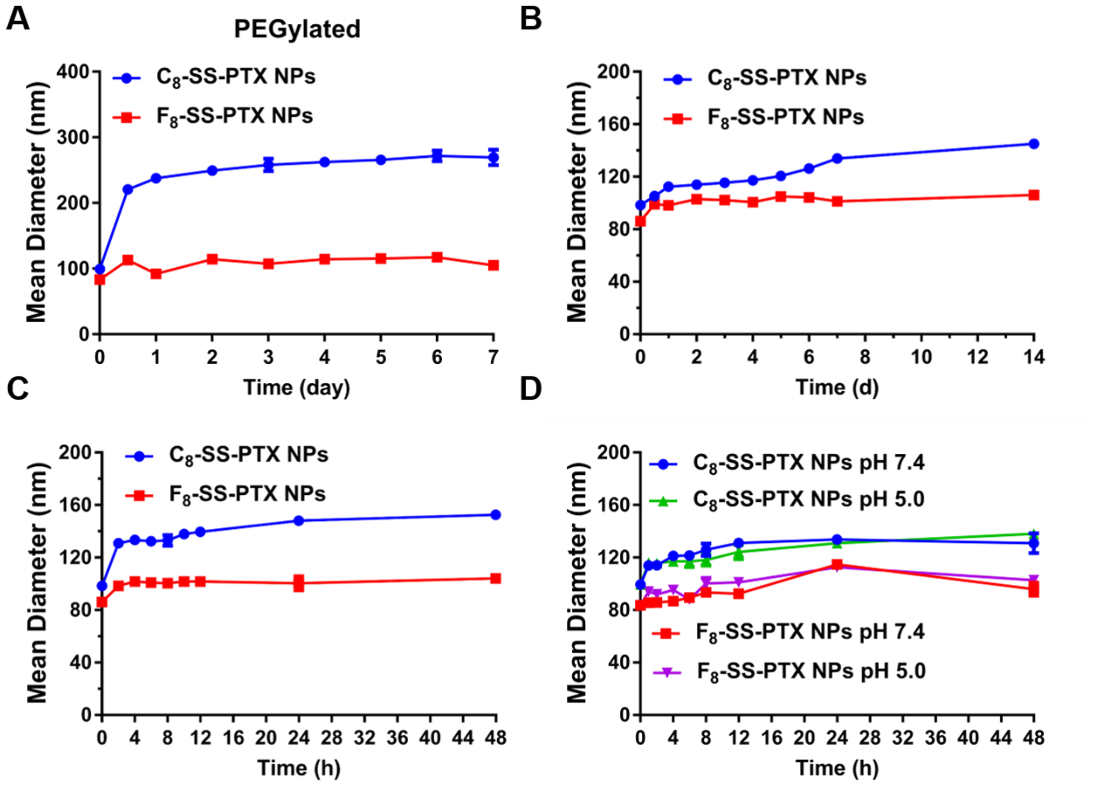

Figure S6. Colloidal stability of PEGylated prodrug nanoassemblies. (A) after storing for 7 d at room temperature; (B) stored at 4 °C for 14 days. (C) incubated in pH 7.4 PBS supplemented with 10% fetal bovine serum (FBS) for 48 h at 37 °C. (D) incubated with culture medium (pH 7.4 and pH 5.0) containing 10% fetal bovine serum (FBS) for 48 h at 37 °C.

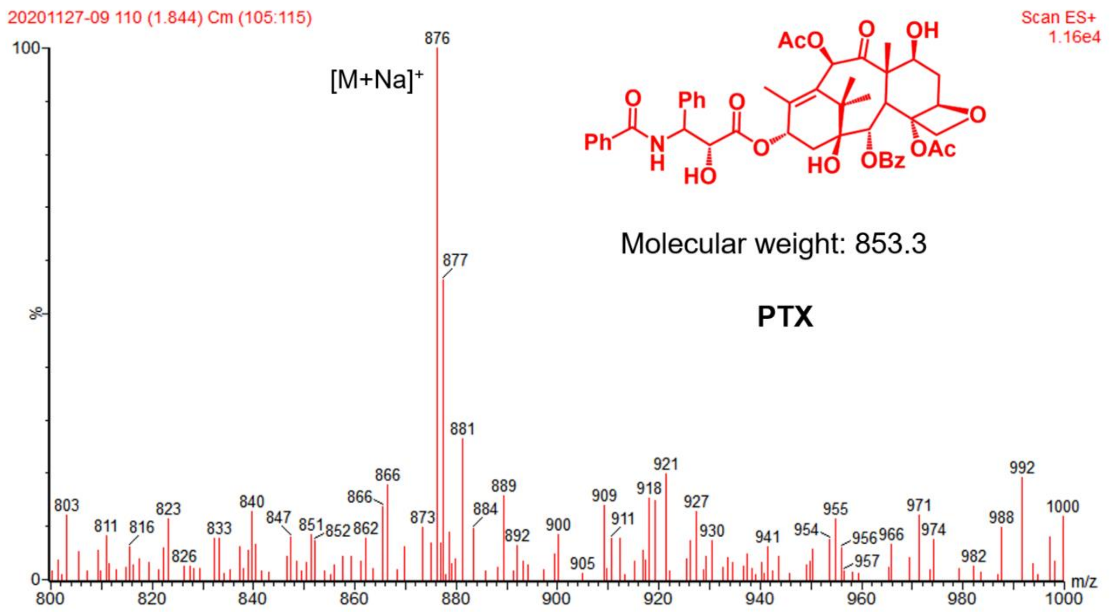

Figure S7. The mass spectrum of eluate at 6.4 min.

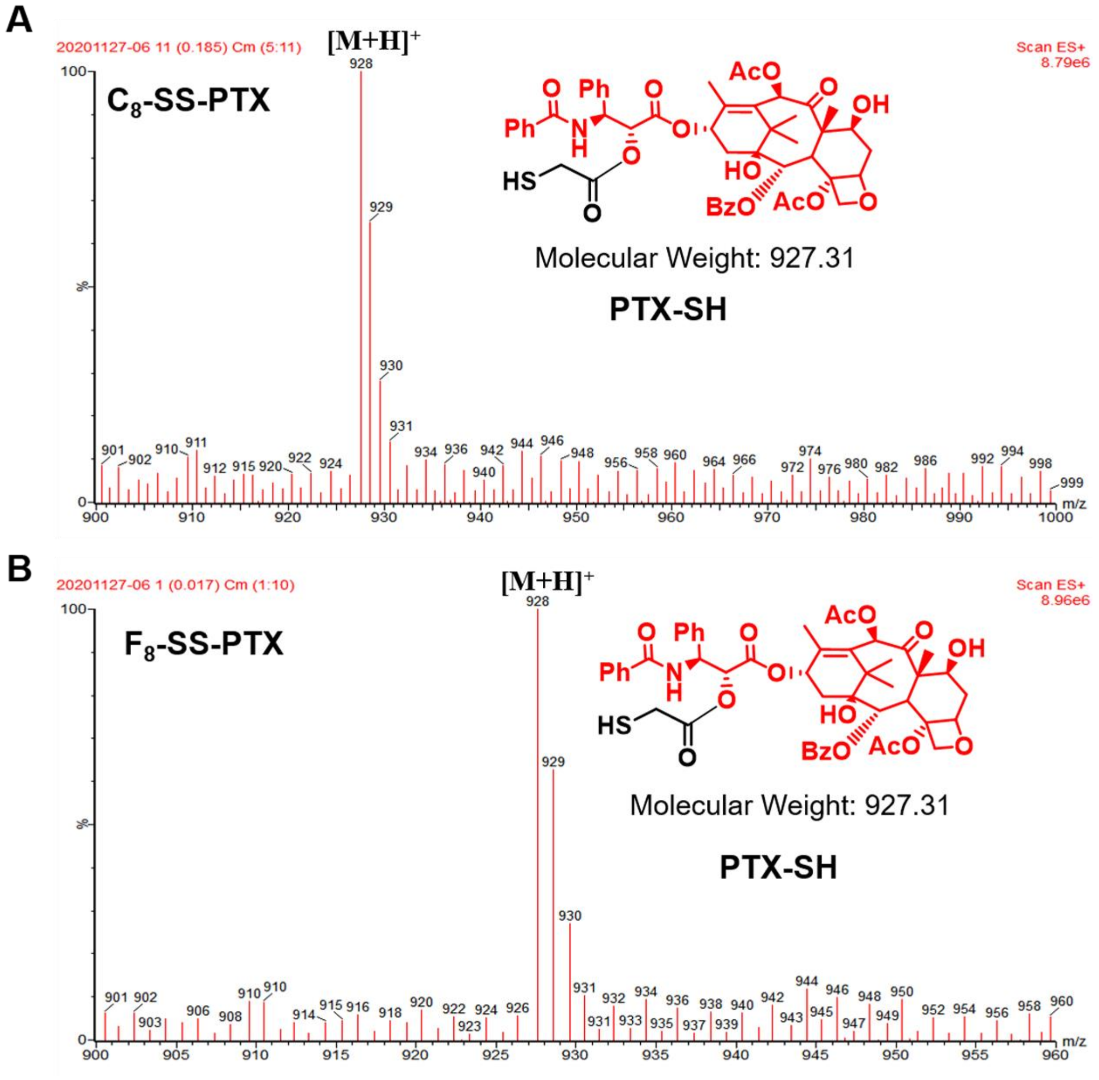

Figure S8. Mass spectra of (A) C_8-SS-PTX and (B) F_8-SS-PTX after incubated with DTT containing release media.

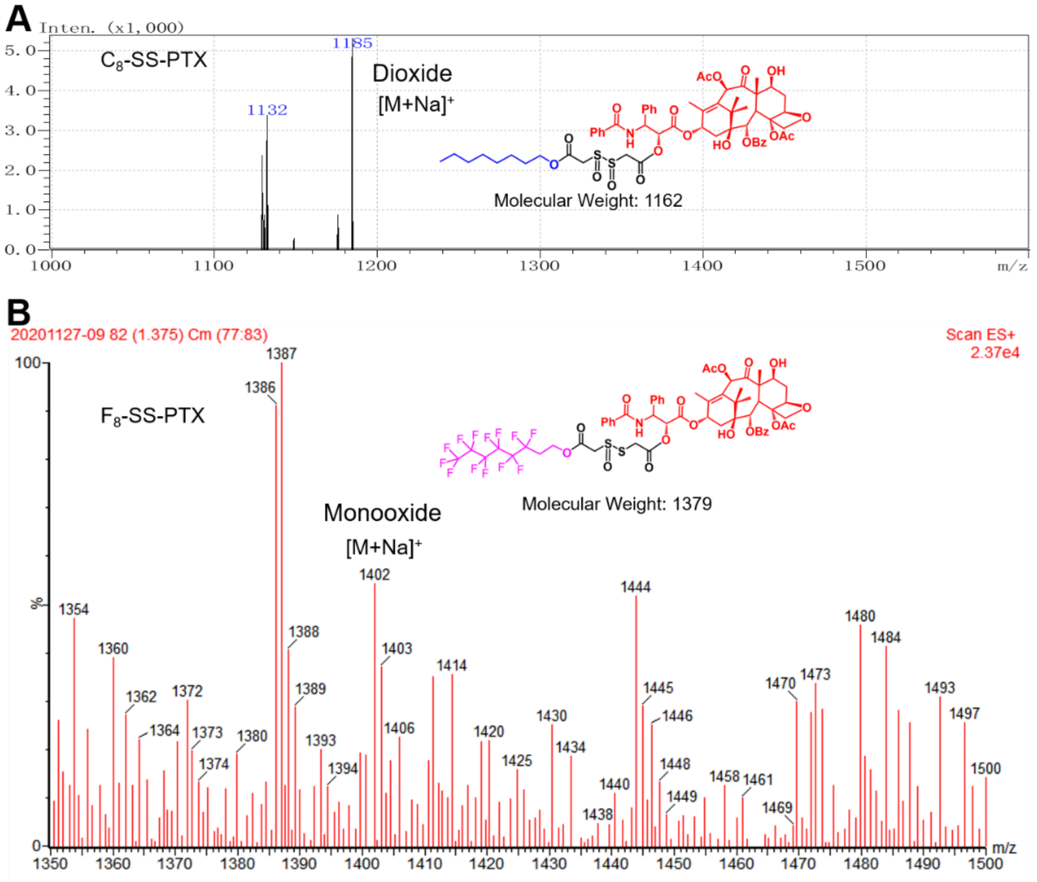

Figure S9. Mass spectra of (A) C_8-SS-PTX and (B) F_8-SS-PTX after incubated with H_2O_2 containing release media.

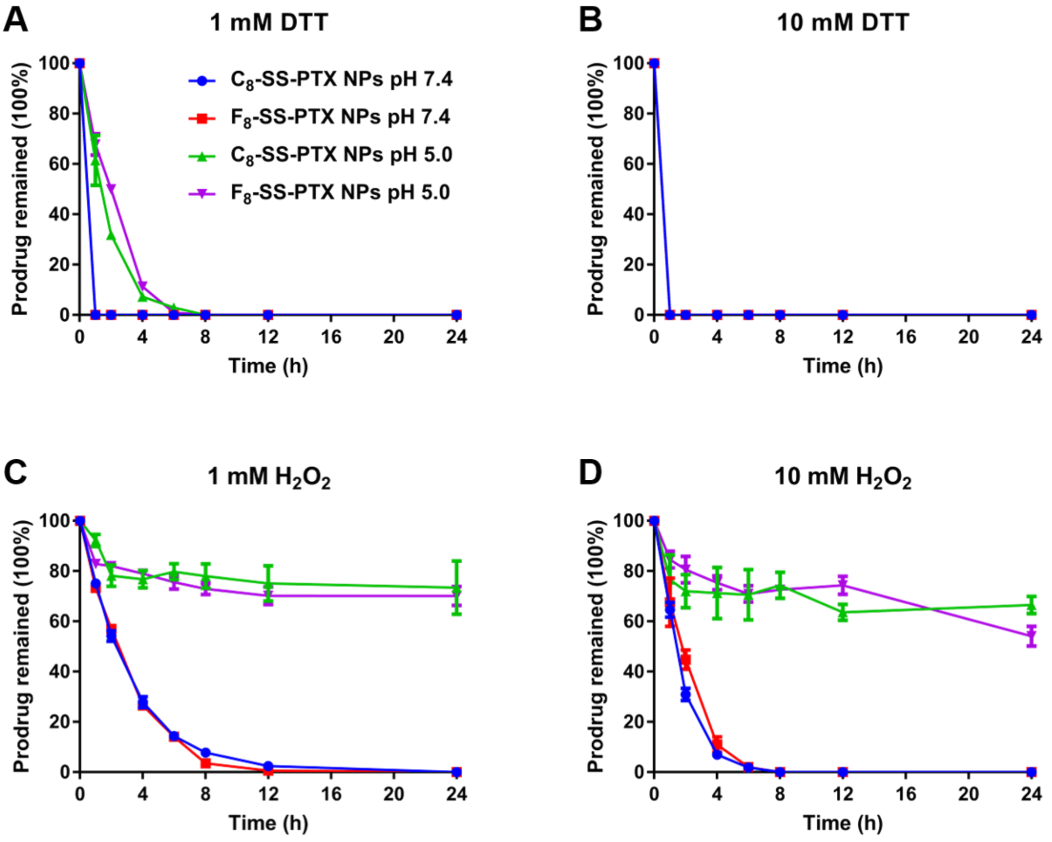

Figure S10. *In vitro* redox-responsive hydrolysis of prodrug nanoassemblies. (A) 1mM DTT; (B) 10 mM DTT; (C) 1mM H_2O_2; (D) 10 mM H_2O_2. Data are presented as the mean ± SD (n=3).

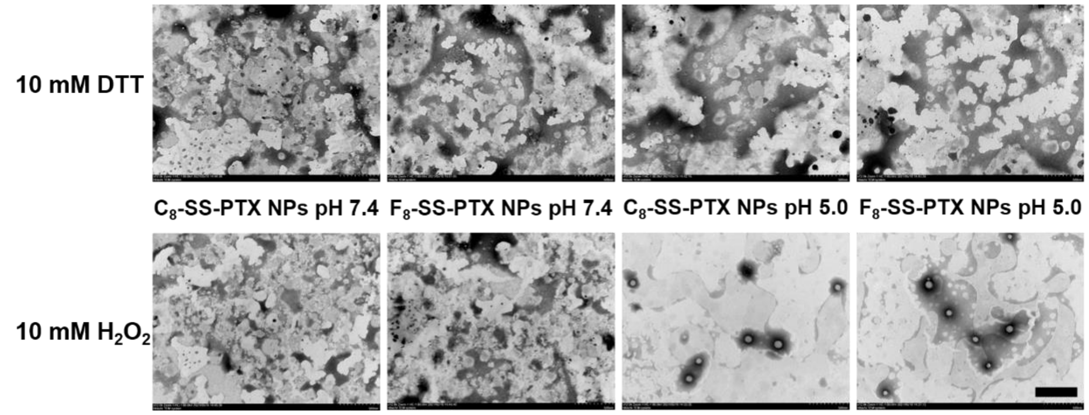

Figure S11 TEM images of prodrug nanoassemblies in the presence of 10 mM DTT (pH 7.4 and pH 5.0) and 10 mM H_2O_2 (pH 7.4 and pH 5.0) for 24 h. The scale bar represents 500 nm.

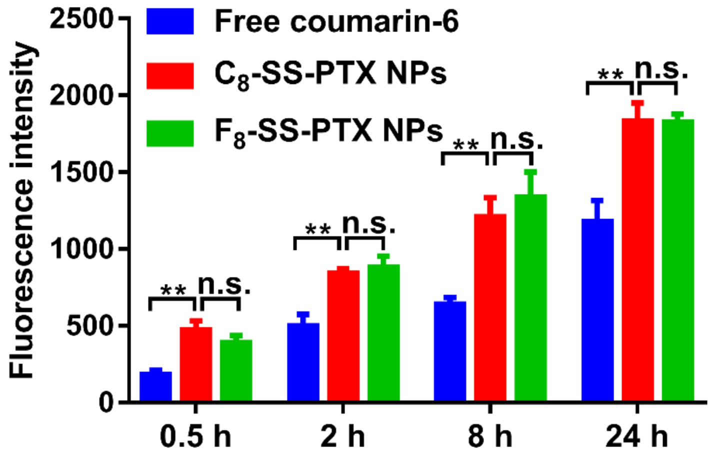

Figure S12. Flow cytometric analyses of CT26 cells after incubation with free coumarin-6 or coumarin-6-labeled prodrug nanoassemblies for 24 h. *$P < 0.05$, **$P < 0.01$, ***$P < 0.001$ by two-tailed Student's t test.

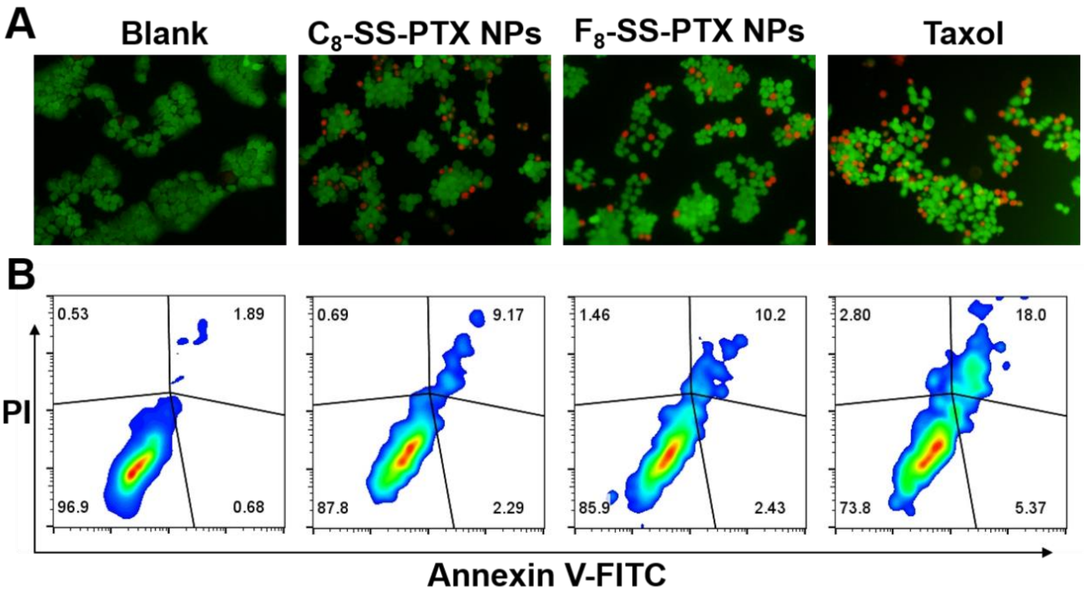

Figure S13. (A) Fluorescence images of Calcein AM (green, live cells) and PI (red, dead cells) co-stained CT26 cells after incubation with blank medium, C8-SS-PTX NPs, F8-SS-PTX NPs and Taxol. (B) Cellular apoptosis assay of Taxol and prodrug nanoassemblies in CT26 cells for 48 h.

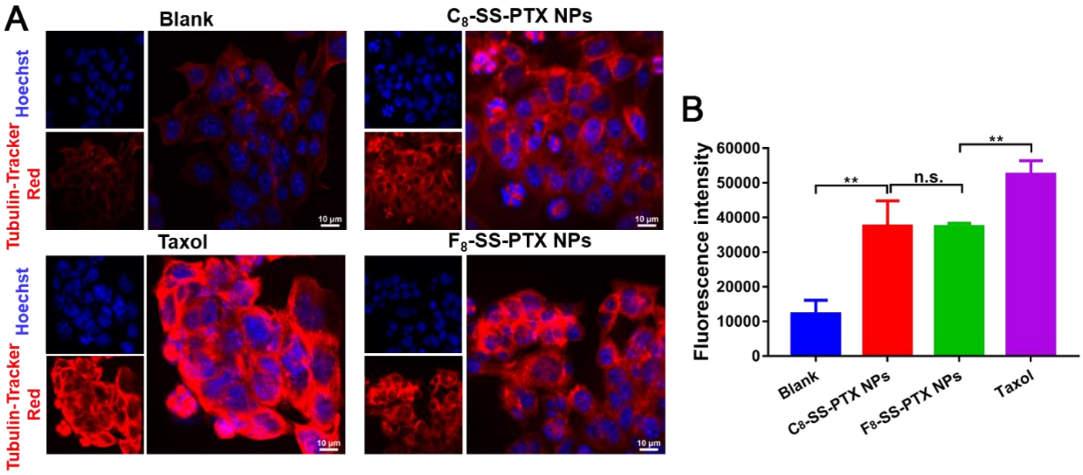

Figure S14. (A) CLSM images of microtubule bundle formation (red) and the nucleus (blue) after treatment with Taxol or prodrug nanoassemblies for 48 h. Scale bar represents 10 μm. (B) The fluorescence intensity in CLSM images analyzed by Image J. **P* < 0.05, ***P* < 0.01, ****P* < 0.001 by two-tailed Student's t test.

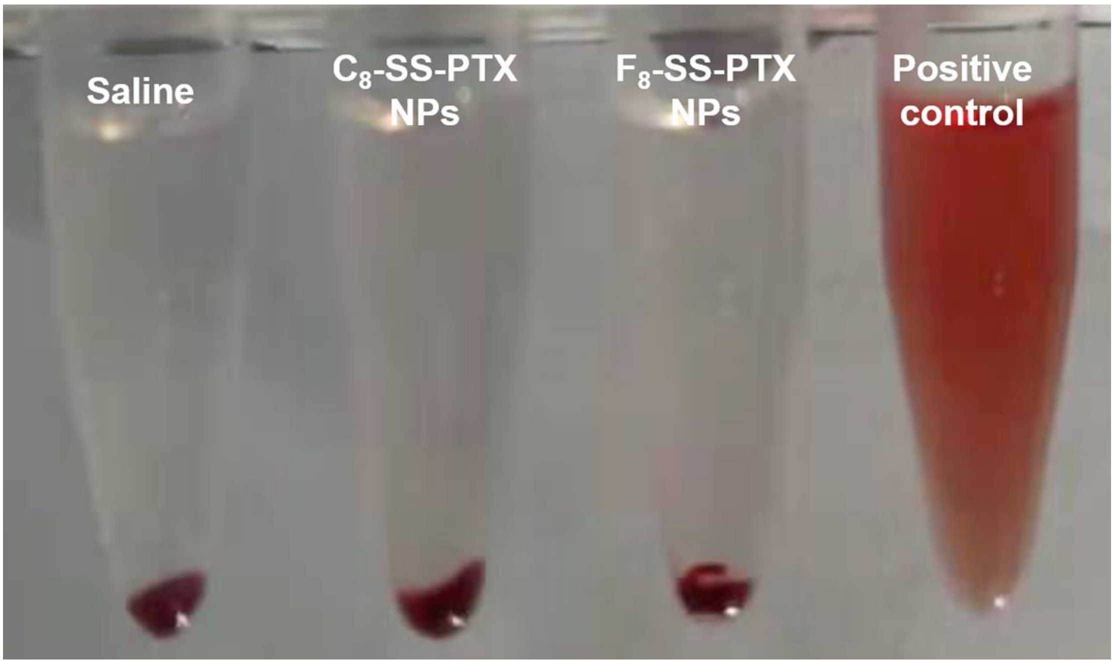

Figure S15. Hemolysis of the prodrug nanoassemblies.

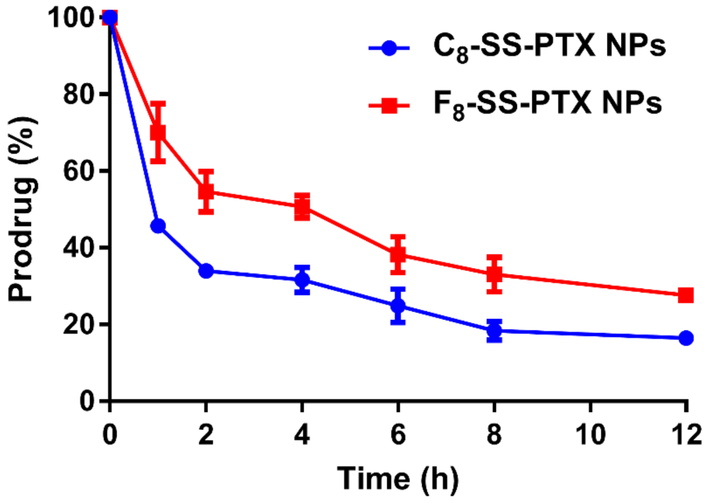

Figure S16. The chemical stability of prodrug nanoassemblies in the fresh rat plasma (n=3).

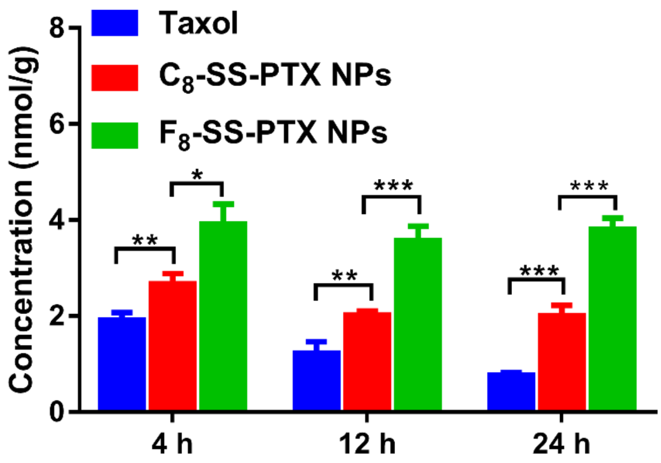

Figure S17. The content of free PTX in the tumor tissues.

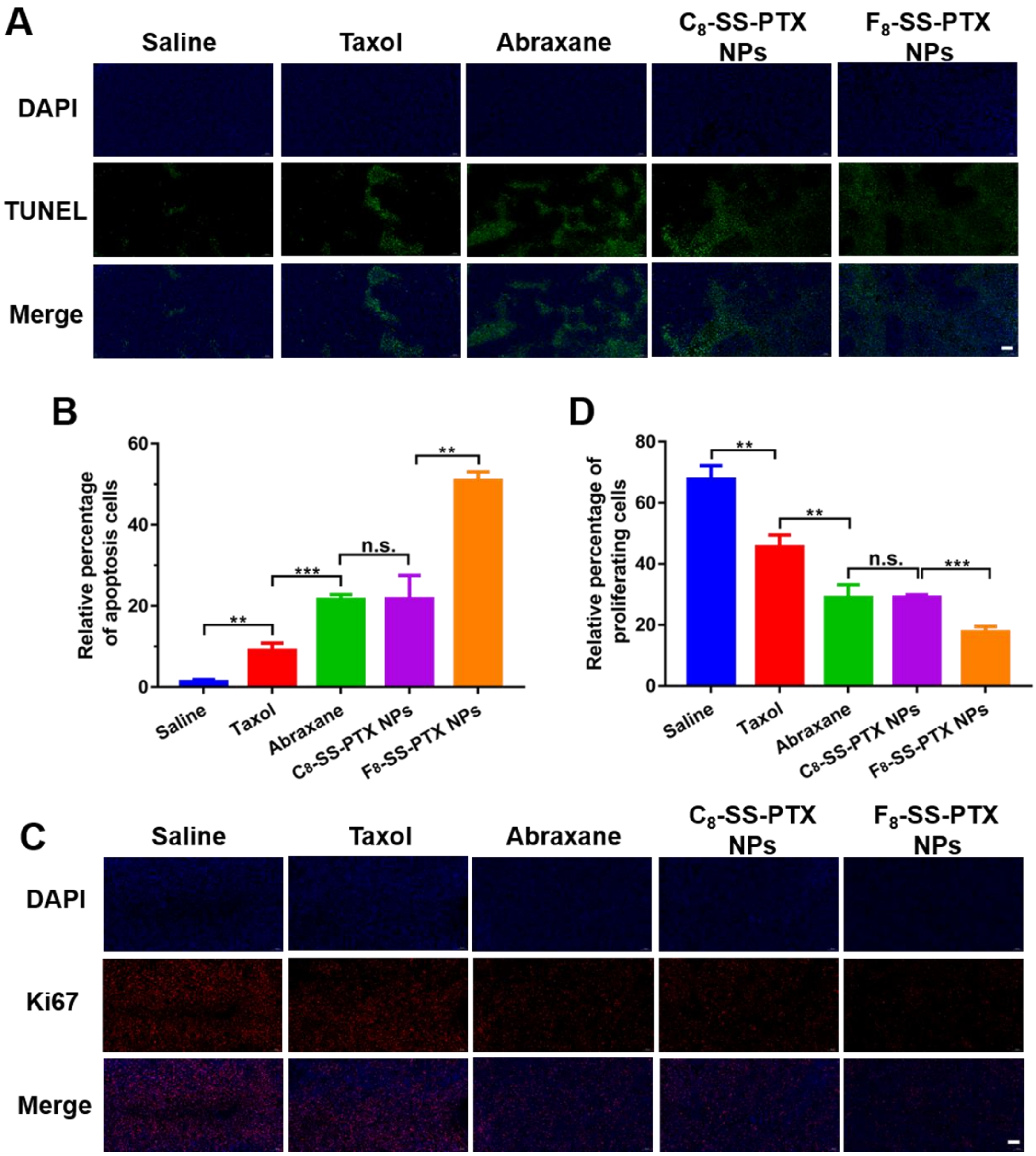

Figure S18. (A) TUNEL assay. CT26 tumor sections were prepared after the last treatment. Scale bar represents 100 μm. (B) Quantification of the relative area (%) of apoptosis cells. (C) Ki67 assay. CT26 tumor sections were prepared after the last treatment. Scale bar represents 100 μm. (D) Quantification of the relative area (%) of proliferating cells. ** $P < 0.01$, ** $P < 0.001$ and **** $P < 0.00001$. Data are presented as mean ± SD (n=3).

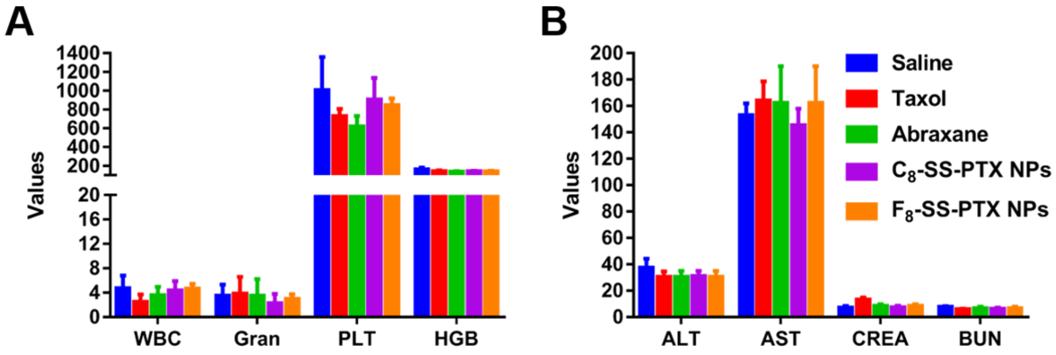

Figure S19. (A) Blood routine examination for CT26 xenograft tumor bearing mice. WBC: white blood cell count (10^9 L^{-1}); Grant: neutrophil count (10^9 L^{-1}); PLT: platelet (10^9 L^{-1}); HGB: hematocrit (g L^{-1}). (B) Hepatorenal function parameters for CT26 xenograft tumor bearing mice. AST: aspartate aminotransferase (U L^{-1}); ALT: alanine aminotransferase (U L^{-1}); BUN: blood urea nitrogen (mmol L^{-1}); CREA: creatinine (μmol L^{-1}). Data are presented as mean ± SD (three independent experiments).

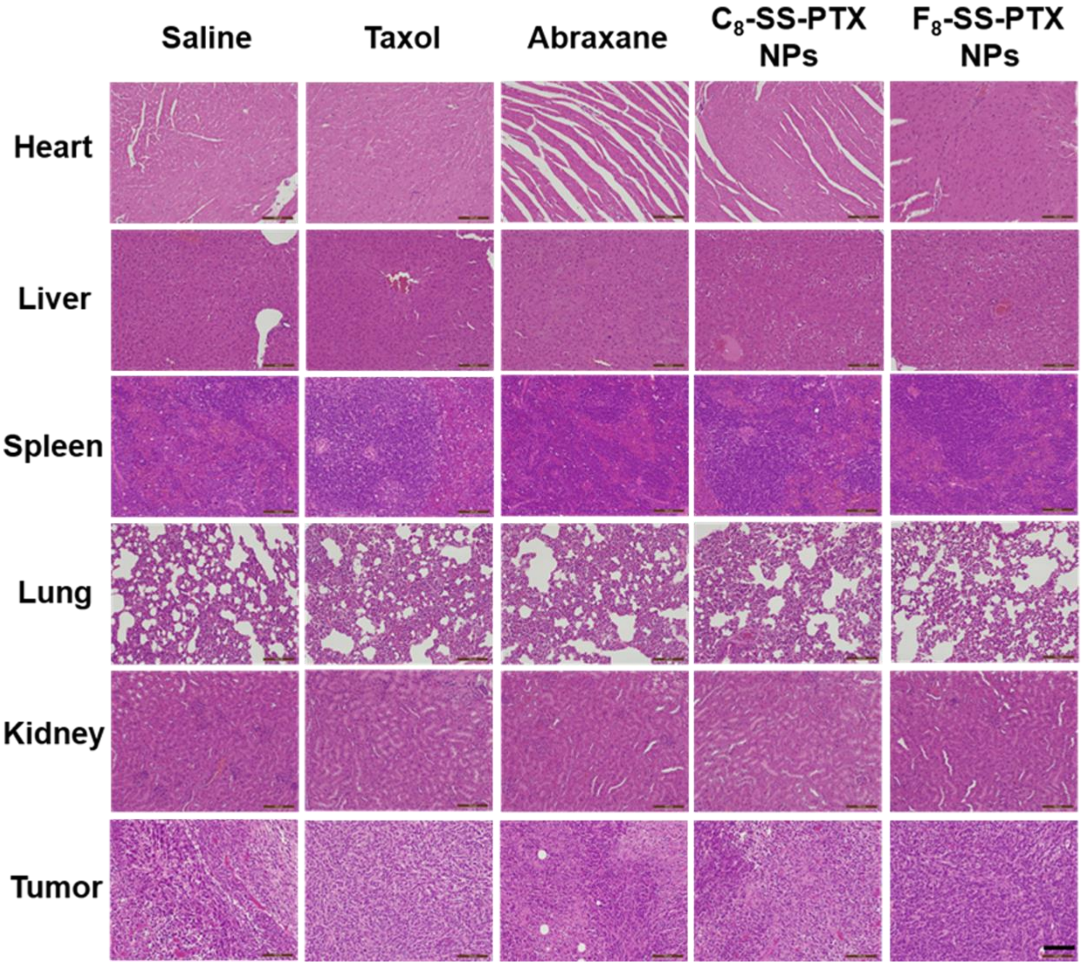

Figure S20. H&E staining of the major organs and tumor of CT26 tumor bearing BALB/c mice. Scale bar represents 100 μm.

Supplementary Tables

Table S1. Characterization of non-PEGylated prodrug nanoassemblies (n=3).

Nanoassemblies	Size (nm)	PDI	Zeta (mV)	DL (w/w)
**C_8-SS-PTX NPs**	107.4±2.61	0.092±0.02	-18.5±0.35	75.8%
**F_8-SS-PTX NPs**	83.02±2.88	0.187±0.022	-6.68±0.80	61.6%

Table S2. Characterization of PEGylated prodrug nanoassemblies (n=3).

Nanoassemblies	Size (nm)	PDI	Zeta (mV)	DL (w/w)
**C_8-SS-PTX NPs**	99.34±1.75	0.098±0.074	-19.9±1.36	60.5%
**F_8-SS-PTX NPs**	83.65±1.45	0.179±0.045	-18.8±1.65	50.1%

Table S3. IC_{50} values (nmol L^{-1}) of Taxol and prodrug nanoassemblies against three tumor cell lines and one normal cell line (n=3).

Cell lines	Taxol	C_8-SS-PTX NPs	F_8-SS-PTX NPs
4T1	23.26	51.74	50.71
A549	5.72	21.38	22.07
CT26	102.5	154.1	129.6
L02	67.59	2706	2598

Table S4. The selectivity index (SI) of prodrug nanoassemblies between normal cells and tumor cells after being incubated for 48 h.

Cell lines	Taxol	C_8-SS-PTX NPs	F_8-SS-PTX NPs
4T1	2.91	52.3	51.23
A549	11.82	126.57	117.72
CT26	0.66	17.56	20.05

Table S5. Pharmacokinetic parameters of Taxol and prodrug nanoassemblies.

Formulations	Determined[a)]	AUC_{0-24h}[b)]	C_{max}[c)]
Taxol	PTX	13.59±1.60	21.36±2.74
C_8-SS-PTX NPs	C_8-SS-PTX	4.71±0.50	15.15±3.06
	PTX	13.44±1.37	17.12±5.08
F_8-SS-PTX NPs	F_8-SS-PTX	91.71±56.92	103.40±67.49
	PTX	14.24±0.71	3.77±1.93

a) Prodrugs and the released PTX were simultaneously determined. b) Area under the plasma concentration-time curve (nmol h mL^{-1}). c) Peak plasma concentration (nmol h mL^{-1}). Data are presented as mean ± SD (n=5).
